# Extracellular vesicle-mediated immune-metabolic crosstalk: research advances in inflammatory regulation and the pathogenesis of metabolic diseases

**DOI:** 10.3389/fimmu.2026.1884606

**Published:** 2026-07-28

**Authors:** Hao-ran Wang, Xue-hui Lv, Yue Sun, Zhi-chao Gu, Meng Lin, Le Guan, Qing-feng Wang, Shi-meng Wang

**Affiliations:** 1 Liaoning University of Traditional Chinese Medicine, Shenyang, China; 2 The Fourth Affiliated Hospital of China Medical University, Shenyang, China

**Keywords:** causality framework, extracellular vesicles, immune–metabolic crosstalk, insulin resistance, macrophage polarization, MASLD, metaflammation, vesiculome

## Abstract

Extracellular vesicles (EVs)—lipid bilayer-enclosed nanoparticles secreted by virtually all cell types—have shifted from being viewed as passive cellular byproducts to active signaling nodes orchestrating inter-cellular and inter-organ communication. Yet the field has accumulated faster than it has integrated: hundreds of EV–cargo–phenotype associations exist as isolated edges of a network whose system-level architecture remains poorly defined. Here we propose the *vesiculome* as an operational framework for that network, resting on three falsifiable axioms: (i) the EV complement of an organism constitutes a network addressable by donor cell × target tissue × cargo class; (ii) cargo composition tracks donor-cell metabolic state in a quantitatively predictable way; and (iii) the integrated balance between pro-inflammatory and pro-resolving vesicle outputs — rather than any single edge — determines the organismal metabolic phenotype. Organized along the chain of EV generation, immune–metabolic interaction, disease mechanism, and translational application, the review synthesizes how this network sustains metabolic homeostasis and how its dysregulation drives metaflammation, the chronic low-grade inflammation underlying obesity, type 2 diabetes mellitus, metabolic dysfunction-associated steatotic liver disease, and atherosclerosis. We dissect two reciprocal arcs of this vesiculome. Polarization-specific EVs from M1/M2 macrophages, Th17 and regulatory T cells, dendritic cells, NK cells, and neutrophils deliver inflammatory or protective cargo to adipose tissue, liver, and pancreas. EVs from adipocytes, hepatocytes, skeletal myocytes, pancreatic β-cells, and intestinal epithelial cells reciprocally reshape the immune microenvironment. Disease arises as a network-level configuration of these arcs rather than as isolated edge failures. We apply a four-tier causality framework to every claim, distinguishing correlational evidence from cargo-depletion and physiological-dose validation. Only miR-155, miR-122, miR-690, and miR-33 currently satisfy the highest tier; most cargoes require systematic experimental escalation before therapeutic translation. We further separate preclinical from clinically validated evidence, and downgrade plant-derived nanovesicles to a methodological cautionary note given non-reproducible cross-kingdom claims. The translational arm evaluates EV-based liquid biopsy biomarkers, native and engineered therapeutic EVs, and mesenchymal stem cell-derived EVs against this framework. We close with a structured catalogue of foundational, causal, and translational knowledge gaps and a priority research agenda built on single-EV multi-omics, *in vivo* tracking, multi-organ organoid-on-chip platforms, and longitudinal human cohorts.

## 1 Introduction

Obesity, type 2 diabetes mellitus (T2DM), metabolic dysfunction-associated steatotic liver disease (MASLD), and atherosclerosis together constitute the largest non-communicable disease burden globally, and their prevalence is rising faster than therapeutic innovation. Two decades of mechanistic work have converged on a shared substrate beneath these conditions: metaflammation, a persistent low-grade immune activation driven by overnutrition and metabolic stress, mechanistically distinct from classical acute inflammation (1). Sustained free fatty acid signaling biases adipose tissue macrophages from the anti-inflammatory M2 state toward the pro-inflammatory M1 state; the resulting TNF-α, IL-1β, and IL-6 output impairs insulin signaling, accelerates hepatic steatosis, and primes vascular inflammation (2). Macrophage polarization is itself coupled to metabolic reprogramming — M1 cells depend on aerobic glycolysis with succinate-driven HIF-1α stabilization, whereas M2 cells rely on fatty acid oxidation and oxidative phosphorylation (3). CD4^+^ T-cell subset differentiation is similarly governed by metabolic substrate availability (4). Immune polarization and metabolic state are therefore not parallel processes but two faces of a single regulatory program.

Despite this conceptual maturity, the molecular vehicles that coordinate immune–metabolic coupling across cells and organs remain ill-defined. Soluble cytokines and circulating metabolites each explain part of the signal, but neither accounts for the combinatorial, cell-of-origin-encoded communication that increasingly characterizes the metaflammatory phenotype. Extracellular vesicles (EVs) — nano- to micrometer-scale lipid bilayer-enclosed particles secreted by virtually every cell type — provide the missing layer. They carry proteins, nucleic acids, lipids, and metabolites in donor-cell-specific composition, and deliver this package to recipient cells without direct contact (5). The lipid bilayer protects cargo from extracellular nucleases and proteases. A single vesicle simultaneously delivers transcriptional regulators, post-transcriptional modulators, and metabolic substrates, enabling parallel rather than serial reprogramming of recipient cells. The field has now matured beyond its descriptive phase: PubMed-indexed EV publications have grown roughly fifty-fold since 2010, more than 350 active clinical trials are ongoing, and the MISEV2023 consensus has consolidated nomenclature and minimum reporting standards (5, 6).

Yet the field has accumulated faster than it has integrated. Hundreds of EV–cargo–phenotype associations now exist as isolated edges of a network whose system-level architecture remains poorly characterized. Three shortcomings have limited translational impact: over-reliance on correlational data and murine models without rigorous causal validation; non-standardized isolation that generates apparent biological discordance reflecting technique rather than biology; and an absence of operational frameworks that distinguish disease-driver EVs from passenger EVs (7). This review responds by proposing the vesiculome as an operational framework. The vesiculome is defined as the total complement of EVs across an organism, organized as an addressable network indexed by donor cell, target tissue, and cargo class. Its cargo composition tracks donor-cell metabolic state in a quantitatively predictable way, and the balance between pro-inflammatory and pro-resolving vesicle outputs — rather than the activity of any single edge — determines the organismal metabolic phenotype. This framing reframes EV research as a question of network topology rather than of cargo enumeration, and it specifies experiments (single-EV multi-omics under defined metabolic perturbations; hub-versus-edge intervention comparison) that render the concept empirically testable.

Throughout this review we apply a four-tier causality framework to every mechanistic claim. Tier 1 denotes correlational data; Tier 2 adds reciprocal EV transfer; Tier 3 adds donor-side genetic loss-of-function; Tier 4 adds *in-vivo* physiological-dose validation. Of the dozens of EV cargoes implicated in metabolic disease, only miR-155, miR-122, miR-690, and miR-33 currently reach Tier 4 (**Table 1**). Most remaining cargoes — including many proposed as biomarkers or therapeutic targets — sit at Tier 1–2 and require cargo-depletion and physiological-dose experiments before robust translation. The sub-stoichiometric cargo problem — the average copy number of any individual miRNA per EV is less than one — further constrains single-vesicle delivery models and is treated as a foundational gap in §7.2.2 (7). We also distinguish preclinical from clinically validated evidence throughout. Robust human cohort data exist for serum and urinary EV miRNA panels in MASLD, T1D, and T2DM, but every native and engineered EV therapeutic discussed in §6 remains preclinical or Phase I in metabolic indications (8). **Table 2** catalogues this distinction explicitly.

**Table 1:** Four-tier causality evidence framework applied to major EV cargoes in immune–metabolic crosstalk.

EV-Borne cargo	Disease context	Correlative (human cohort)	Donor-side genetic KO/depletion	Reciprocal transfer sufficient	In-vivo physiological dose validated	Final tier	References
miR-155 (M1-ATM)	Obesity/IR	✓ multiple cohorts	✓ Mir155^-^/^-^	✓ ATM-EV swap	✓	Tier 4	(29)
miR-122 (hepatocyte ASGR1^+^ EV)	MASLD/NASH	✓ (AUC 0.84; n > 300)	✓	✓	✓	Tier 4	(64)
miR-33a/b (vasc.)	Atherosclerosis	✓	✓ antagomir	✓	✓	Tier 4	(89)
miR-690 (M2-BMDM)	Obesity (therapy)	✗ (no human)	✓	✓	✓ (mouse)	Tier 3 (mouse-only)	(30)
miR-34a (adipocyte)	Obesity/IR	✓	✓	partial	✗	Tier 3	(51)
TRAIL-EV (hepatocyte)	NASH	✓	✓ ROCK1 inhibition	✓	✗	Tier 3	(59)
Ceramide C16:0 (hepatocyte)	NASH	✓	✓ STARD11/IRE1α	✓	✗	Tier 3	(62, 63)
CXCL10 (hepatocyte EV)	NASH	✓	partial	✓	✗	Tier 2–3	(60)
miR-192 (ASGR1^+^ EV)	MASLD biomarker	✓ (n > 300)	✗	✗	✗	Tier 2 (biomarker)	(64)
miR-20b-5p (plasma)	T2DM	✓ (n = 145)	✗ (siRNA in vitro only)	✗	✗	Tier 2	(79)
miR-130a/miR-145 (urinary)	Diabetic nephropathy	✓ (case-control n = 89)	✗	✗	✗	Tier 2	(84)
miR-141-3p↓ (adipose→liver)	Obesity hepatic IR	partial	✓	✓	✗	Tier 3	(65)
mtdsRNA (hepatocyte)	ASH; NASH	✗	✓ TLR3^-^/^-^	✓	✗	Tier 2 (alcohol model)	(33)
β-cell autoantigen EV (GAD65, ZnT8)	T1D	✓ (human islets)	partial	✓	✗	Tier 2	(67, 69, 70)
miR-19b-3p (tubular)	Diabetic nephropathy	partial	partial	✓	✗	Tier 2	(85)
miR-503 (endothelial)	T2DM vascular	✓	✗	partial	✗	Tier 2	(83)
Engineered PD-L1 EV	T1D (therapy)	✗	—	✓ (mouse)	✗	Tier 2 (mouse-only)	(72)
MyomiRs (miR-133a, miR-1)	Exercise metabolic protection	small cohorts	✗	partial	✗	Tier 1–2	(65, 66)
GELN miRNA (mdo-miR7267-3p)	Colitis/gut homeostasis	✗	✗	✓ (mouse)	✗	Tier 1 (contested)	(102-104)
MSC-EV (HucMSC)	T2D/MASLD (therapy)	Phase I safety only	—	✓ (mouse)	✗	Tier 2–3 (mouse); Phase I	(97, 98, 101)

Each cargo claim is evaluated against four independent evidence criteria: correlative association in human cohorts; donor-side genetic depletion abolishing the phenotype; reciprocal EV transfer recapitulating the phenotype; and *in-vivo* validation at physiological cargo concentrations. The final tier reflects the highest criterion satisfied with all lower-tier criteria also met. Tier 4 (green) cargoes (miR-155, miR-122, miR-690 in mouse only, miR-33) currently anchor the field’s strongest mechanistic claims. Most cargoes remain at Tier 1–3 and require systematic experimental escalation before therapeutic translation (§7.2). References correspond to citation numbers in the main manuscript reference list. Symbols: ✓, satisfied; ✗, not yet demonstrated; partial, partially satisfied or under active investigation; —, not applicable.

**Table 2:** Catalogue of human and clinical evidence for EV-based translational applications in metabolic disease.

Translational application	Modality	Highest tier	Cohort size/phase	Primary endpoint	Translation status	Section	References
ASGR1^+^ EV miR-122/miR-192 for MASLD histological stratification	Biomarker	Tier 4	Two cohorts, n > 300	AUC ≈ 0.84 (NAFL vs NASH)	Validation phase	§5.2, §6.1	(64)
Plasma exosomal miR-20b-5p for T2DM insulin resistance	Biomarker	Tier 3	Cross-sectional, n = 145	HOMA-IR correlation	Validation	§5.1, §6.1	(79)
Urinary exosomal miR-130a/miR-145 for early diabetic nephropathy	Biomarker	Tier 2	Case-control, n = 89	Pre-microalbuminuria detection	Discovery	§5.3, §6.1	(84)
Circulating leukocyte-derived EV abundance for coronary atherosclerosis	Biomarker	Tier 2–3	Multiple cohorts, n > 800	Plaque-burden correlation	Discovery → Validation	§5.4, §6.1	(87, 88)
Plasma exosomal miR-375 for T1D β-cell mass monitoring	Biomarker	Tier 2	Small cohorts	β-cell mass surrogate	Discovery	§4.4, §6.1	(94)
Bone marrow MSC-EV in COVID-19 metabolic inflammation	Native EV therapy	Phase I	n ≈ 24	Safety	Phase I completed (adjacent indication)	§6.3	(101)
HucMSC-Exos in T2DM (multi-axis: peripheral + hepatic + islet)	Native EV therapy	Tier 2–3	Mouse only	Glucose homeostasis	Preclinical	§6.3	(97, 98)
ADSC-EV in HFD-induced obesity (M2 polarization rescue)	Native EV therapy	Tier 3	Mouse only	~27.8% IR improvement	Preclinical	§6.2	(32)
M2-BMDM miR-690 EV in HFD obesity	Native EV therapy	Tier 3	Mouse only	Restored insulin sensitivity	Preclinical	§6.2	(30)
Engineered PD-L1 EV for T1D immune tolerance	Engineered EV	Tier 2	Mouse only	Autoreactive T-cell suppression	Preclinical	§4.4, §6.3	(72)
Engineered antigen-HLA EV with CD80/PD-L1 for antigen-specific T-cell control	Engineered EV	Tier 2	Mouse only	Antigen-specific T-cell modulation	Preclinical	§4.4, §6.3	(73)
miR-33 antagomir EV strategy for atherosclerosis	Engineered EV	Tier 3–4	Mouse + small primate	ABCA1/G1↑; plaque regression	Preclinical → IND planning	§5.4, §6.3	(89)
Ginger-derived nanovesicles (GELNs) for gut barrier/colitis	PDNV (plant)	Tier 1	Mouse + ex-vivo human organoid	Barrier function; AHR-IL-22 axis	Preclinical (contested)	§6.3	(102-104)
Hollow-fiber bioreactor/3D spheroid GMP scale-up	Manufacturing	—	Process development	50–100× yield gain	Pilot scale; no consensus protocol	§6.5	(107, 108)
TFF + SEC GMP purification workflow	Manufacturing	—	Process development	Reproducible yield + purity	Consolidating standard	§6.5	(107, 113, 114)
FDA HCT/P vs. 21 CFR Part 351 EV classification	Regulatory	—	Framework	Pathway assignment	Draft guidance 2023	§6.5	—
EMA ATMP/GTMP/sCTMP EV classification	Regulatory	—	Framework	Pathway assignment	Active framework	§6.5	—
Single-EV multi-omics (MASEV; cryo-ET; single-EV miRNA-seq)	Foundational tool	—	Method development	Sub-population resolution	Methodology priority	§7.1, §7.5	(112, 123, 124)
Multi-organ organoid-on-chip with immune co-culture	Foundational tool	—	In vitro model	Inter-organ EV crosstalk	Methodology priority	§7.5	(9, 125, 126)
Longitudinal prospective cohorts (MASLD priority)	Cohort design	—	Discovery → Validation	EV reference intervals; predictive models	High priority	§7.3, §7.5	—

Translational maturity is heterogeneous across modalities. Biomarker applications (top block) have reached Tier 3–4 evidence with cohort-validated performance in MASLD stratification, T2DM insulin resistance, and diabetic nephropathy. Native EV and MSC-EV therapeutic applications (middle block) have completed Phase I safety evaluation only in adjacent indications and remain preclinical for metabolic disease. Engineered EVs and plant-derived nanovesicles remain entirely preclinical. Manufacturing, regulatory, and methodological dimensions (bottom block) are framework-level rather than tier-graded. References correspond to citation numbers in the main manuscript reference list; “—” indicates framework-level items without specific primary citations. AUC, area under the curve; ATMP, advanced therapy medicinal product; GMP, good manufacturing practice; GTMP, gene therapy medicinal product; HCT/P, human cell, tissue, and cellular and tissue-based product; HFD, high-fat diet; IND, investigational new drug; MASEV, multiplexed analysis of single extracellular vesicles; PDNV, plant-derived nanovesicle; sCTMP, somatic cell therapy medicinal product; SEC, size-exclusion chromatography; T2DM, type 2 diabetes mellitus; TFF, tangential flow filtration.

The review is organized along a four-stage chain: EV generation and architecture (§2); the two reciprocal arcs of immune–metabolic interaction, in which immune-cell EVs reprogram metabolic tissues (§3) and metabolic-cell EVs reprogram immune populations (§4); disease mechanisms as configurations of the same dysregulated vesiculome rather than as isolated edge failures, covering obesity, MASLD/NASH, T2DM, and atherosclerosis (§5); and translational applications spanning biomarkers, native and engineered EVs, MSC-EVs, and plant-derived nanovesicles, each evaluated against the four-tier framework (§6). §7 catalogues foundational, causal, and translational knowledge gaps and proposes the experimental program — single-EV multi-omics, *in-vivo* tracking, organoid-on-chip platforms, and longitudinal human cohorts — required to convert the vesiculome from a conceptual claim into a falsifiable research program (9). §8 closes with synthesis. By repositioning EVs from a list of cargo–phenotype pairs to an integrated information layer of immune–metabolic crosstalk, this review aims to provide both a theoretical framework and a tractable experimental agenda for next-generation precision diagnosis and intervention in metabolic disease.

## 2 Extracellular vesicle generation, architecture, and recipient engagement

**Figure 1:**
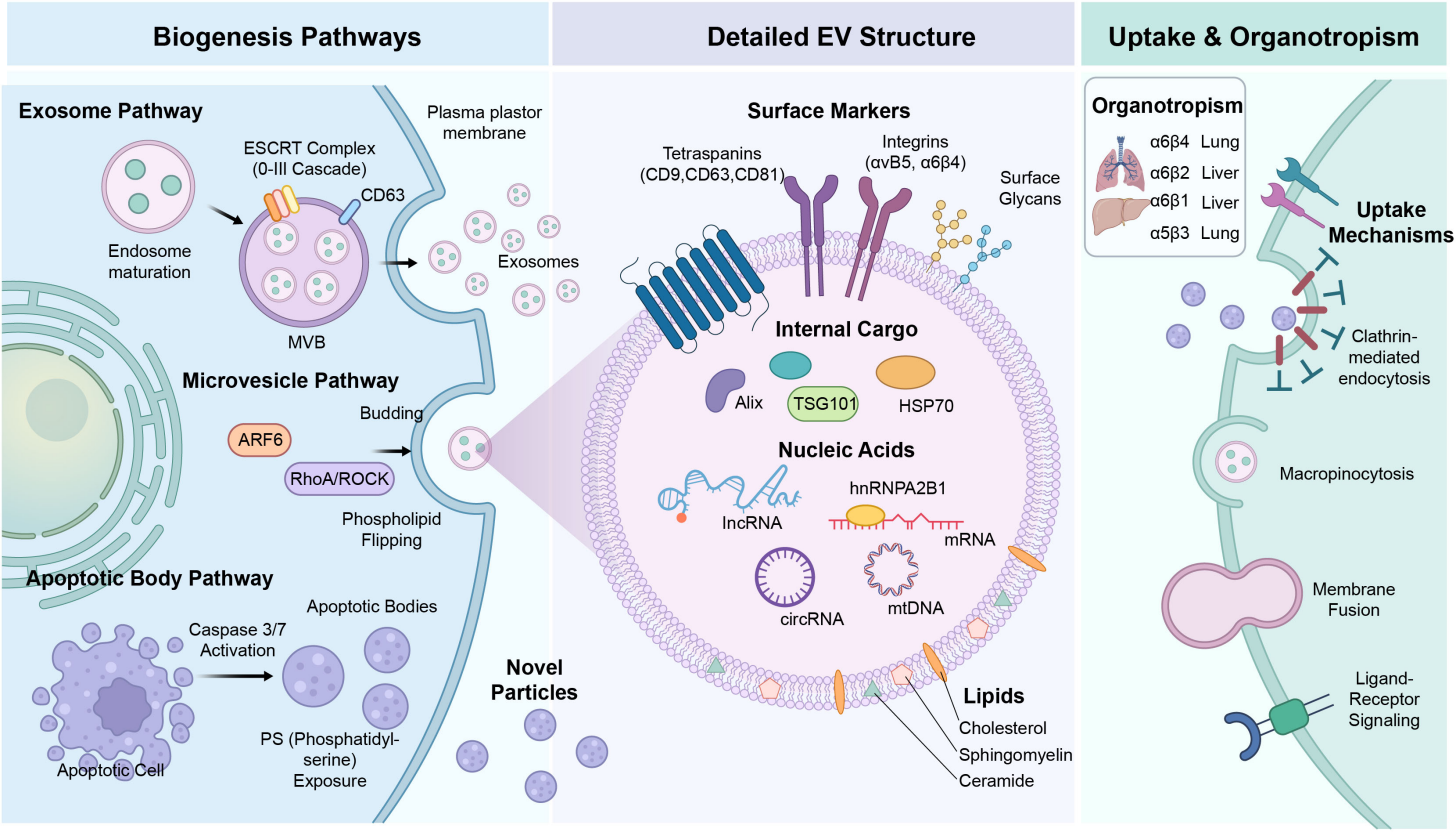
The biogenesis, cargo architecture, and intercellular communication of extracellular vesicles (EVs). The schematic depicts the complete life cycle of EVs. (Left, Biogenesis Pathways) Three principal biogenesis routes are illustrated. Exosomes (30–150 nm) originate from the endolysosomal system: early endosomes mature into multivesicular bodies (MVBs), whose limiting membrane invaginates to form intraluminal vesicles (ILVs) through ESCRT-0/I/II/III-dependent or ESCRT-independent (nSMase2/ceramide and tetraspanin-microdomain) machinery; MVB–plasma membrane fusion releases ILVs as exosomes. Microvesicles (100–1,000 nm) bud directly outward from the plasma membrane under the control of ARF6, RhoA/ROCK, and phospholipid (phosphatidylserine) flipping. Apoptotic bodies (>1,000 nm) are released downstream of caspase-3/7 activation and membrane blebbing during programmed cell death, with surface phosphatidylserine (PS) exposure serving as the “eat-me” signal. (Center, Detailed EV Structure) Surface markers include the tetraspanin family (CD9, CD63, CD81), tropism-determining integrins, and surface glycans. Luminal cargo comprises canonical EV-identity proteins (Alix, TSG101, HSP70), selectively loaded nucleic acids (miRNA, lncRNA, circRNA, mRNA, mtDNA) — with hnRNPA2B1 shown as a representative RNA-binding sorter — and characteristic lipids (cholesterol, sphingomyelin, ceramide). Non-vesicular nanoparticles (exomeres/supermeres) are indicated as the “novel particles” category that lies below the classical EV size range. (Right, Uptake & Organotropism) Integrin-dependent organotropism directs EVs to specific organs. EVs enter recipient cells through clathrin-mediated endocytosis, macropinocytosis, direct membrane fusion, or ligand–receptor signaling. EV, extracellular vesicle; ESCRT, endosomal sorting complexes required for transport; MVB, multivesicular body; ILV, intraluminal vesicle; nSMase2, neutral sphingomyelinase 2; ARF6, ADP-ribosylation factor 6; RhoA, Ras homolog family member A; ROCK, Rho-associated kinase; PS, phosphatidylserine; CD, cluster of differentiation; TSG101, tumor susceptibility gene 101; HSP70, heat shock protein 70; hnRNPA2B1, heterogeneous nuclear ribonucleoprotein A2/B1; miRNA, microRNA; lncRNA, long non-coding RNA; circRNA, circular RNA; mtDNA, mitochondrial DNA.

This section establishes the operational EV biology required for the immune–metabolic biology in §3–§5. We treat the well-established elements concisely and foreground only the features that constrain the network claims that follow. **Figure 1** summarizes this biogenesis-to-engagement sequence.

### 2.1 From cellular waste to an information layer

EV research has been defined by a conceptual inversion. Plasma procoagulant particles described in the 1940s, the “exosomes” identified in the 1980s as a mechanism for reticulocyte receptor disposal, and the micro vesicles and apoptotic bodies subsequently characterized were once interpreted as cellular waste. Contemporary evidence has overturned this view. Phospholipid bilayer-enclosed particles spanning nanometer to micrometer scales—released by virtually every cell type studied—are active vehicles of inter-cellular and inter-organ communication. They transfer a structured payload of proteins, nucleic acids, lipids, and metabolites that reprograms recipient cell behavior (10).

Two properties distinguish EV-mediated signaling from soluble signaling. The lipid bilayer shelters cargo from extracellular nucleases and proteases, conferring stability that free miRNAs and unprotected proteins cannot match. A single vesicle simultaneously delivers transcriptional regulators, post-transcriptional modulators, and metabolic substrates, enabling parallel rather than serial reprogramming (11). Stability plus combinatorial capacity is precisely what positions EVs as the information layer of the vesiculome proposed in §1.

### 2.2 Cargo architecture as the proximate determinant of EV bioactivity

EV taxonomy has historically been built around biogenesis route and morphology. The bioactive cargo a vesicle carries, however, is the proximate determinant of its effect on a recipient cell (12). We therefore foreground cargo here and treat biogenesis in §2.3 as the machinery that generates cargo specificity.

A defining observation of EV biology is that the small-RNA content inside an EV does not mirror the small-RNA content of its parental cell. Cargo loading is therefore an active, selective process, not passive entrapment (13). RNA-binding proteins constitute the molecular machinery of this selectivity. hnRNPA2B1 recognizes a GGAG-containing EXOmotif gated by SUMOylation. YBX1 sorts miR-223 through phase-separated cytosolic condensates (14). Beyond miRNA, EVs carry lncRNAs and circRNAs; circRNAs are particularly enriched because their covalently closed backbone resists exonuclease attack. EVs also traffic double-stranded DNA, including mitochondrial DNA fragments that engage cytosolic cGAS-STING in recipient cells to trigger type I interferon and inflammatory programs—a mechanism increasingly invoked in paracrine immunomodulation (15).

Quantitative proteomic surveys catalogue thousands of EV-associated proteins. Syntenin-1 has emerged as the most abundant exosomal protein and is endorsed by MISEV2023 alongside the canonical tetraspanins CD9, CD63, and CD81 and the ESCRT-associated proteins Alix and TSG101 (6, 16). EV-borne receptor tyrosine kinases can be transferred horizontally to install ligand-responsive signaling in recipient cells, and vesicular protein signatures alone now distinguish multiple human cancers (17). The metabolic enzyme LDHA is preferentially carried in the non-vesicular supermere fraction, linking extracellular signaling directly to recipient cell metabolic state (18).

EV lipidomes diverge systematically from the plasma membrane of the parental cell. Phosphatidylserine (PS), sphingomyelin, cholesterol, and ceramide are all enriched, raising the proportion of liquid-ordered domains and biophysically rigidifying the vesicle bilayer (19). Externalized PS provides the molecular handle recognized by TIM and TAM scavenger receptors during uptake, doubling as an “eat-me” signal and a high-affinity tether to recipient cells (19, 20). EV membranes also carry sphingosine kinase activity that locally generates sphingosine-1-phosphate, engaging G-protein-coupled receptors with consequences for vascular biology and inflammation.

Cargo composition is not fixed. It is continuously sculpted by the physiological and pathological state of the donor cell, and this responsiveness is precisely what underpins liquid-biopsy strategies (12). Hypoxia shifts EV cargo toward pro-angiogenic factors; inflammation remodels both protein and miRNA loads on short timescales; chemotherapy induces tumor cells to release EVs enriched with drug-efflux pumps and resistance-conferring miRNAs (21). EV content is a real-time readout of donor cell biology rather than a static signature. This responsiveness is the empirical anchor of Axiom (ii) (§1) — state-dependent cargo reconfiguration — and provides its operational testability: any chronic metabolic perturbation should imprint a reproducible reconfiguration of EV cargo that is measurable by paired single-EV multi-omics (§7.5) and that reverses upon perturbation withdrawal. Whether this prediction holds at single-vesicle resolution remains an open empirical question and a priority direction for the field.

### 2.3 Biogenesis pathways generate cargo specificity

EV subtypes arise through three routes, each contributing a characteristic combination of size, cargo bias, and release kinetics. Live-cell imaging has refined what was once a rigid taxonomy. Some “small” EVs once attributed to endosomal export in fact bud directly from the plasma membrane, and rigorous subtype assignment now requires biogenesis-tracing evidence rather than size alone (22).

The endosomal route produces the small EV population conventionally called exosomes (30–150 nm). Early endosomes mature into multivesicular bodies; the limiting membrane invaginates inward to generate intraluminal vesicles; microtubule-dependent trafficking delivers a subset of these bodies to the plasma membrane, where SNARE- and Rab-regulated fusion releases the vesicles. Membrane sculpting is executed by the endosomal sorting complexes required for transport (ESCRT) acting sequentially through cargo recognition, membrane deformation, and scission, supported by VPS4-driven disassembly. Parallel ESCRT-independent routes operate through neutral sphingomyelinase 2-generated ceramide, which provides the conical lipid geometry needed for negative membrane curvature, and through tetraspanin-enriched microdomains that template specific transmembrane cargoes (12, 23, 24).

Microvesicles (100–1000 nm) bud outward from the plasma membrane. Their formation requires phospholipid asymmetry breakdown, partial actin cortex remodeling, and small-GTPase signaling through ARF6, which activates a phospholipase D–ERK relay and contractile scission at the vesicle neck (24). Because small ectosomes and large exosomes overlap in size, and conventional population-level isolation cannot separate them, size alone is unreliable for subtype attribution (6). Apoptotic bodies (>1000 nm) form during programmed cell death. Caspase-mediated cleavage of ROCK I drives actin–myosin contraction and produces the characteristic membrane blebbing; their PS-rich surface ensures phagocytic clearance and tissue homeostasis (25).

Beyond the canonical taxonomy, asymmetric flow field-flow fractionation has revealed non-vesicular nanoparticles smaller than classical EVs. Exomeres (~35 nm) are enriched in metabolic enzymes and glycolytic factors and lack canonical tetraspanin markers. Supermeres (~25–30 nm) carry disease-associated proteins including TGFBI and LDHA together with a distinctive extracellular RNA spectrum (18, 26). The extracellular particle landscape therefore extends below the canonical small-EV size range; biomarker discovery should not be confined to membrane-bounded species. The diversity of biogenesis routes is the mechanistic substrate underlying Axiom (i) (§1) — the addressable-network claim. Distinct donor-cell programs generate identifiable vesicle classes that index donor identity, polarization state, and metabolic context; whether these classes can in fact be resolved at single-vesicle resolution across the full immune–metabolic compartment is the empirical question to which the experimental program of §7.1 and §7.5 is addressed.

### 2.4 Recipient cell engagement and the limits of methodology

The biological consequence of any EV depends not only on what it carries but on which cell internalizes it and on whether the cargo escapes the endolysosomal system to reach a functional compartment. This recipient-side biology is the rate-limiting step for EV-based interventions.

Recipient selection is biased by combinatorial surface signatures. Integrin heterodimers direct tissue-level homing; αvβ5 targets vesicles to hepatic Kupffer cells, while α6β4 and α6β1 combinations drive pulmonary distribution (27). CD47 on the EV surface engages SIRPα on phagocytes to transmit a “don’t eat me” signal that delays clearance—a property now exploited in therapeutic EV engineering (28). Externalized PS binds TIM and TAM family receptors to anchor vesicles prior to uptake, with central importance for immune-cell engagement (20). Internalization proceeds through clathrin-mediated endocytosis, macropinocytosis, and direct membrane fusion, with the chosen route influencing whether cargo reaches an endolysosomal dead end or a productive cytosolic compartment.

Two methodological constraints qualify every claim derived from current EV studies and recur as foundational gaps in §7. First, EV isolation methods are not interchangeable. Differential ultracentrifugation, size-exclusion chromatography, polymer precipitation, immunoaffinity capture, and microfluidic capture each enrich different sub-populations, and the resulting baseline heterogeneity is the single largest source of cross-study discordance. MISEV2023 codifies minimum reporting standards but does not eliminate this variability (6). Second, the mean copy number of any individual miRNA per single EV is sub-stoichiometric—typically less than one copy per vesicle. Single-vesicle delivery models are therefore mechanistically untenable for most miRNA claims, and population-level effects must arise from cooperative delivery across many vesicles. Resolving this requires single-vesicle multi-omics with absolute quantification, treated as a priority direction in §7.4 (7).

These constraints are not peripheral. They define the rigor with which any vesiculome-level claim — including those developed in §3–§5 — must be evaluated. They operationally anchor Axiom (iii) (§1) — the network-balance principle: given baseline isolation heterogeneity and sub-stoichiometric cargo, single-edge causal claims are systematically under-powered, whereas network-balance claims (ratios of pro-inflammatory to pro-resolving vesicles; configurational shifts across the immune–metabolic compartment) are robust to these confounds. This is why §3–§5 frame disease as a configuration of the network rather than as an aggregation of independent cargo–phenotype pairs. .

## 3 The immune-cell arc of the vesiculome: how immune EVs reprogram metabolic tissues

**Figure 2:**
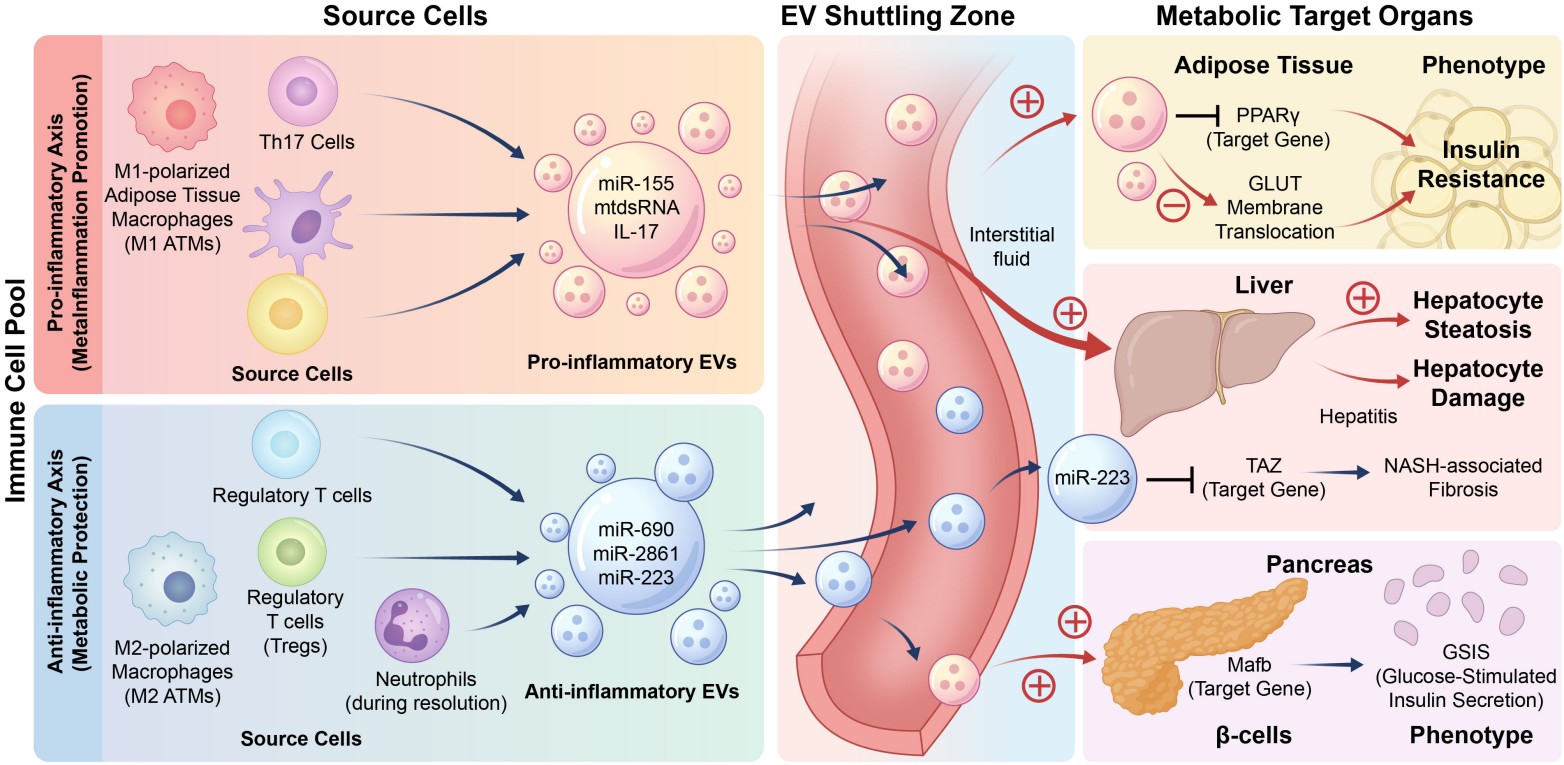
The dual roles of immune cell-derived EVs in meta-inflammation and metabolic homeostasis. The schematic illustrates the bipartite EV signaling network linking the immune cell pool to metabolic target organs. (Pro-inflammatory Axis — Meta-inflammation Promotion): M1-polarized adipose tissue macrophages (M1 ATMs), Th17 cells, and other inflammatory effector cells secrete pro-inflammatory EVs enriched in miR-155, mitochondrial double-stranded RNA (mtdsRNA), and IL-17. After transit through the interstitial fluid and circulation (“EV Shuttling Zone”), these EVs act on (i) adipose tissue, where miR-155 suppresses PPARγ and impairs GLUT4 membrane translocation, driving insulin resistance; and (ii) the liver, where they promote hepatocyte steatosis and hepatocyte damage characteristic of NASH. (Anti-inflammatory Axis — Metabolic Protection): M2-polarized macrophages (M2 ATMs), regulatory T cells (Tregs), and pro-resolving neutrophils release protective EVs carrying miR-690, miR-2861, and miR-223. miR-223 delivered to hepatocytes suppresses TAZ and attenuates NASH-associated fibrosis. EVs reaching pancreatic β-cells engage Mafb and preserve glucose-stimulated insulin secretion (GSIS). The dynamic equilibrium between the two axes dictates the metabolic fate of insulin-target organs. EV, extracellular vesicle; ATM, adipose tissue macrophage; M1/M2, classically/alternatively activated macrophages; Th17, T helper 17 cell; Treg, regulatory T cell; mtdsRNA, mitochondrial double-stranded RNA; IL-17, interleukin-17; PPARγ, peroxisome proliferator-activated receptor gamma; GLUT, glucose transporter; TAZ, transcriptional coactivator with PDZ-binding motif; NASH, non-alcoholic steatohepatitis; Mafb, MAF bZIP transcription factor B; GSIS, glucose-stimulated insulin secretion.

This section develops the first of the two reciprocal arcs of the immune–metabolic vesiculome. Polarization-specific EVs from macrophages, T cells, dendritic cells, NK cells, and neutrophils transmit donor-cell identity, polarization state, and metabolic context to recipient cells in adipose tissue, liver, and pancreas. Across these populations a consistent dichotomy emerges. Vesicles from M1 macrophages, Th17 cells, and mature dendritic cells form a pro-inflammatory axis that converges on PPARγ suppression, M1 polarization amplification, and Th17 expansion in metabolic target tissues. Vesicles from M2 macrophages, regulatory T cells, and resolution-phase neutrophils form a counter-balancing pro-resolving axis that converges on KLF4-licensed M2 polarization and Mafb-stabilized β-cell function. The balance between these axes—not the absolute output of either—determines the inflammatory tone of metabolic tissues (**Figure 2**; **Table 3**). We treat each cellular source in turn, then close with an integrative appraisal of evidence strength.

**Table 3:** EV cargo, source, target, and disease map across the immune–metabolic vesiculome.

#	EV source/state	Key cargo	Target cell	Biological effect	Disease context	Section	Evidence tier	References
1	Adipocyte (obese, hypertrophic)	miR-34a; ceramides; free cholesterol	ATM; hepatocyte	KLF4↓ → M1 polarization; PP2A → Akt↓; lipid-driven ATM differentiation	Obesity, IR, MASLD	§4.1, §5.1	Tier 3	(3, 51, 52)
2	ATM (M1-polarized)	miR-155; mtdsRNA; TNF-α; IL-1β	Adipocyte; hepatocyte; myocyte	PPARγ↓ across tissues; insulin signaling↓; cross-tissue IR propagation	Obesity, IR	§3.1, §5.1	Tier 4	(29)
3	ATM (M2-polarized)/BMDM (M2)	miR-690; STAT3 protein; Arg1 inducers	Insulin-target cells	Nadk↓ → restored insulin sensitivity; ~27.8% IR improvement	Obesity (therapy)	§3.1, §6.2	Tier 3 (mouse)	(30-32)
4	Th17 cell	IL-17; mtdsRNA (via hepatocyte relay)	Hepatocyte; macrophage	IL-17 amplification; NASH inflammation	NASH, ASH	§3.2	Tier 2	(33, 34)
5	Treg cell	miR-2861; miR-223; OSM	Monocyte; HSC (TAZ↓); adipose progenitor	Wound healing; NASH fibrosis attenuation; adipose homeostasis	T2D, NASH	§3.2	Tier 2	(36-38, 44)
6	Hepatocyte (lipotoxic, DR5-active)	TRAIL; CXCL10; ceramide C16:0; mtDNA	Macrophage; HSC	IL-1β/IL-6↑; HSC activation; pro-fibrotic cascade	NASH → fibrosis	§4.2, §5.2	Tier 3	(59-63)
7	Hepatocyte (ASGR1^+^, lipotoxic)	miR-122; miR-192	Circulating (biomarker pool)	Distinguishes NAFL from NASH (AUC ≈ 0.84, n > 300)	MASLD/NASH (biomarker)	§5.2, §6.1	Tier 4	(64)
8	Skeletal myocyte (exercise-induced)	miR-133a; miR-1; irisin	Hepatocyte; adipocyte; macrophage	Hepatic insulin sensitivity↑; adipose browning; M2 polarization	Obesity, MASLD (protective)	§4.3, §5.5	Tier 1–2	(65, 66)
9	β-cell (cytokine-stressed)	GAD65; ZnT8; proinsulin; MHC-I↑	APC; autoreactive CD8^+^ T cell	Break of islet immune privilege; T-cell activation	T1D, T2D (β-cell loss)	§4.4, §5.3	Tier 2	(67, 69, 70]
10	β-cell (immunomodulatory baseline)	miR-7; miR-21; miR-146; miR-155; miR-375	Islet immune cells	Immune-tolerance maintenance; β-cell mass readout	T1D (biomarker)	§4.4, §6.1	Tier 2	(68, 94)
11	Endothelial cell (hyperglycemic)	ICAM-1; VCAM-1; miR-503; miR-155; miR-126↓	Monocyte; recipient endothelium	Vascular inflammation; impaired VEGF/repair	T2DM vascular complications	§5.3	Tier 2	(83)
12	Tubular epithelial cell (hyperglycemic)	miR-19b-3p	Macrophage	M1 polarization → renal inflammation	Diabetic nephropathy	§5.3	Tier 2	(85)
13	Retinal pigment epithelium (high glucose)	VEGF	Retinal endothelium	Pathological neovascularization	Diabetic retinopathy	§5.3	Tier 2	(86)
14	Foam cell (cholesterol-loaded)	oxLDL lipids; S100A8/A9; MMPs	VSMC; intraplaque macrophage	Plaque destabilization; foam cell amplification	Atherosclerosis	§5.4	Tier 3	(87, 88)
15	Platelet (shear-activated)	P-selectin; GPIbα; arachidonic acid metabolites	Intraplaque macrophage	Pro-thrombotic + pro-inflammatory signaling	Atherosclerosis	§5.4	Tier 2	(87)
16	Macrophage/vascular cell (athero)	miR-33a/b	Macrophage cholesterol efflux machinery	ABCA1/G1↓ → impaired RCT; foam cell positive feedback	Atherosclerosis	§5.4	Tier 4	(89)
17	Intestinal epithelial cell (IEC)	miRNAs; tolerogenic antigens	Lamina propria DC; immune cells	Tolerance induction; metabolic interorgan signaling	Obesity, MASLD (homeostatic)	§4.5, §5.5	Tier 1–2	(74-76)
18	Gut bacteria (BEV; dysbiosis)	LPS; bacterial DNA	Kupffer cell; hepatocyte; ATM	cGAS-STING/TLR activation → metaflammation amplification	MASLD, T2DM, obesity	§4.5, §5.5	Tier 2–3	(77, 78)
19	MSC (HucMSC therapeutic)	Trophic miRNAs; growth factors	β-cell; hepatocyte; myocyte	β-cell apoptosis↓; AMPK↑; multi-axis metabolic rescue	T2D, MASLD (therapy)	§6.3	Tier 2–3 (mouse); Phase I only	(97, 98, 101)
20	Ginger PDNV (plant-derived)	mdo-miR7267-3p	Lactobacillaceae; gut epithelium	I3A↑ → AHR → IL-22↑ → barrier↑ (cross-kingdom claim contested)	Colitis (preclinical)	§6.3	Tier 1 (cautionary)	(102-104)

Evidence tiers: T1, correlative only; T2, + reciprocal transfer; T3, + donor-side genetic loss-of-function; T4, + in vivo physiological-dose validation (see **Table 1** and §5.6). Color coding: green, Tier 4; blue, Tier 3; yellow, Tier 2; orange, Tier 1. Symbols used in **Table 3**: ↑ indicates an increase, upregulation, or activation; ↓ indicates a decrease, downregulation, or inhibition; → indicates a directional relationship, meaning “leads to,” “promotes,” or “results in” the subsequent biological effect or process. References correspond to citation numbers in the main manuscript reference list. ATM, adipose tissue macrophage; APC, antigen-presenting cell; ASH, alcoholic steatohepatitis; BEV, bacterial extracellular vesicle; BMDM, bone-marrow-derived macrophage; DC, dendritic cell; GELN, ginger-derived exosome-like nanoparticle; HSC, hepatic stellate cell; HucMSC, human umbilical cord mesenchymal stem cell; I3A, indole-3-carboxaldehyde; IEC, intestinal epithelial cell; IR, insulin resistance; MASLD, metabolic dysfunction-associated steatotic liver disease; MMP, matrix metalloproteinase; MSC, mesenchymal stem cell; NAFL, non-alcoholic fatty liver; NASH, non-alcoholic steatohepatitis; PDNV, plant-derived nanovesicle; RCT, reverse cholesterol transport; T1D/T2D, type 1/type 2 diabetes; VSMC, vascular smooth muscle cell.

### 3.1 Macrophage-derived EVs as the central hub of metabolic polarization

Macrophages are the most numerous immune population in metabolic organs and the most extensively characterized source of immune-cell EVs. The M1/M2 polarization axis is itself a metabolic phenotype: M1 cells run aerobic glycolysis with succinate-driven HIF-1α stabilization, while M2 cells depend on fatty acid oxidation and oxidative phosphorylation (3). Polarization state is propagated across the tissue not only by cytokines but by EVs whose cargo encodes the metabolic identity of the parental cell.

M1-polarized adipose tissue macrophages (M1-ATMs) release EVs enriched in miR-155, mtdsRNA, and a set of pro-inflammatory cytokines including TNF-α and IL-1β (29). miR-155 silences PPARγ across hepatocytes, myocytes, and adipocytes alike, degrading insulin signaling and providing a unified molecular explanation for the cross-tissue character of obesity-related insulin resistance. Reciprocal vesicle transplantation between lean and obese mice transmits the donor’s metabolic phenotype to the recipient (29). Together with concordant human cohort data and Mir155^-^/^-^ rescue, miR-155 currently satisfies the highest tier of causal evidence (Tier 4: correlative + reciprocal transfer + donor-side knockout + physiological-dose validation).

M2-polarized macrophages provide the counter-arc. Bone marrow-derived M2 exosomes carry miR-690, which targets Nadk in insulin-responsive cells, suppresses inflammation, and restores glucose homeostasis when administered to obese mice (30). The same M2 polarization is propagated by adipose-derived stem cell exosomes, which deliver activated STAT3 protein to recipient macrophages, transactivate arginase-1, and bias the tissue population toward an anti-inflammatory phenotype (31, 32). In diet-induced obese animals, this single intervention improves insulin sensitivity by approximately 27.8%, attenuates adipose inflammatory infiltration, and reduces hepatic steatosis. miR-690 reaches Tier 3 evidence (causal in mouse), but no human cohort data are yet available; this is the central gap separating M2-EV therapy from clinical translation.

The two arcs are not independent. M1-EVs delivered to adipocytes silence PPARγ; M2-EVs delivered to the same adipocytes restore it. Polarization is therefore a dynamic, vesicle-mediated equilibrium rather than a static phenotype. Progressive M2 attrition during obesity tips this equilibrium asymmetrically toward inflammation, providing a vesiculome-level explanation for why adipose inflammatory tone becomes increasingly difficult to reverse as the disease advances (31). The same vesicles are released into the circulation and reach the liver, skeletal muscle, and pancreas, propagating the polarization signal beyond the tissue of origin. Macrophage-derived EVs therefore constitute the principal hub of the immune-cell arc—a network node whose dysregulation, more than any single cytokine, determines the metabolic phenotype.

### 3.2 T lymphocyte-derived EVs and the Th17/treg vesicular equilibrium

T cell-derived EVs operate on a slower timescale than macrophage EVs but carry comparable functional weight in adaptive immune memory. The Th17/Treg balance—long established as a determinant of metaflammation through soluble cytokine signaling—is recapitulated and amplified at the vesicular level.

Th17 cells release EVs carrying IL-17 and Th17-lineage markers. In alcoholic hepatitis, hepatocyte exosomes packaged with mitochondrial double-stranded RNA engage Toll-like receptor 3 on recipient hepatocytes to induce IL-17 production, amplifying Th17-type inflammation in a manner that links nucleic acid danger signals directly to adaptive immune output (33). The immunopathology of non-alcoholic steatohepatitis follows the same logic. IL-17 secreted by Th17 cells promotes intrahepatic inflammatory responses and serves as a pivotal cytokine linking metabolic injury to hepatic fibrosis (34). Visceral adipose tissue Treg numbers are reduced in obesity, and disruption of the JAZF-1/PPARγ pathway impairs Treg differentiation; the resulting Treg/Th17 imbalance exacerbates metabolic injury in insulin target tissues (35).

The pro-resolving arc of T cell EVs derives from CD4^+^CD25^+^FOXP3^+^ regulatory T cells. Treg-derived EVs enriched in miR-2861 are taken up by monocytes and reshape recipient gene expression to promote diabetic wound healing (36, 37). Cord blood Treg-EVs target monocytes and reduce local inflammatory signaling in metabolic tissue repair models. Visceral adipose Tregs also secrete oncostatin M, which negatively regulates adipose progenitor cell differentiation; conditional knockout of oncostatin M or its receptor in Treg cells produces overt insulin resistance, confirming that Treg paracrine signaling is required for adipose immune homeostasis (38). Functional heterogeneity within the Treg compartment further refines this picture: at least two functionally distinct Treg subpopulations exist in visceral adipose, and their composition shifts with age and diet (39). Obesity disrupts Treg cholesterol homeostasis and impairs their immune-suppressive function—a mechanism that defines a candidate therapeutic target for restoring Treg activity (40).

Most claims in this section currently sit at Tier 2 evidence (correlative plus reciprocal transfer). Donor-side genetic depletion of Treg-EV cargo (e.g., miR-2861 conditional knockouts) has not yet been performed in the metabolic context, and the field has been slow to test whether Treg-EVs are necessary, rather than sufficient, for metabolic protection. This is a tractable experimental gap.

### 3.3 Dendritic cell EVs as gatekeepers of adaptive immune activation

Dendritic cells bridge innate and adaptive immunity, and their maturation state directly determines the immunogenicity of their secreted EVs. In NAFLD progression, hepatic dendritic cells shift toward an immunogenic mature phenotype under lipid overload. High-lipid-laden dendritic cells stimulate NK and NKT cells to produce large amounts of TNF-α, IFN-γ, IL-2, and IL-6, while low-lipid dendritic cells are nearly non-immunogenic and instead promote Treg generation and immunological tolerance (41).

Mature dendritic cell EVs carry MHC-II/peptide complexes and high levels of co-stimulatory molecules CD80 and CD86. These vesicles directly activate antigen-specific CD4^+^ T cells and drive Th17 differentiation, accelerating inflammatory progression in NASH (34). The therapeutic counterweight comes from mesenchymal stem cell-derived EVs, which simultaneously suppress Th17 and M1 macrophage proliferation while promoting Treg and M2 expansion—a multi-axis immune correction that defines the conceptual basis for EV-mediated immune reprogramming therapy in metabolic disease (42). Dendritic cell EV biology is currently the least quantified arc of the immune-cell vesiculome. Even single-cell-resolution profiling of metabolic-context dendritic cell EVs remains rare, and **Tier 1** correlative evidence dominates the literature.

### 3.4 Innate lymphoid and granulocyte EVs in metaflammation

Natural killer cells are abundant innate lymphocytes in the liver and participate in hepatic inflammation regulation through EV-mediated communication. Hepatocyte-derived EVs reprogram NK cell proliferation, cytotoxic activity, and cytokine secretion through miRNA transfer, modulating the target specificity of NK cell-mediated killing (43). NK cell function in NAFLD is stage-dependent. During the fibrosis resolution phase, NK cells exert pro-reparative functions by eliminating activated hepatic stellate cells; during active inflammation, the same cells contribute to hepatocyte injury. The directionality of NK-EV output across these stages has not been systematically mapped, and current data sit at Tier 1–2.

Neutrophil-derived EVs occupy a unique position because their function reverses across the inflammation lifecycle. Activated neutrophils release EVs loaded with neutrophil extracellular trap fragments, myeloperoxidase, and elastase, amplifying tissue injury at the inflammatory peak. During the resolution phase, the same cell type secretes EVs enriched in miR-223 and specialized pro-resolving lipid mediators including resolvin D1 and protectin D1 (44). These resolution-phase neutrophil EVs are taken up by hepatic macrophages, where miR-223 targets the TAZ pathway to attenuate NASH-associated fibrosis. Neutrophil-EV directionality therefore tracks the temporal phase of the inflammatory response rather than the cellular identity of the producer—a property that current sampling strategies (cross-sectional, peak-inflammation-biased) systematically miss. This temporal switching is the clearest empirical example of why disease-stage-resolved vesiculome profiling, rather than steady-state characterization, is needed for accurate causal inference (developed as a priority direction in §7).

### 3.5 Critical appraisal and integration

The vesiculome’s immune-cell arc is best summarized as two opposing axes whose balance—not whose absolute outputs—determines metabolic phenotype. The pro-inflammatory axis aggregates M1-ATM, Th17, mature dendritic cell, and activation-phase neutrophil EVs; it converges on PPARγ suppression, Th17 expansion, and macrophage M1 amplification in adipose tissue, liver, and pancreas. The pro-resolving axis aggregates M2-ATM, Treg, tolerogenic dendritic cell, and resolution-phase neutrophil EVs; it converges on KLF4-licensed M2 polarization, oncostatin M-mediated adipose homeostasis, and Mafb-stabilized β-cell function. Disease progression in obesity, MASLD, and T2DM corresponds to progressive imbalance—loss of the pro-resolving arc and gain of the pro-inflammatory arc—rather than to failure of any single edge of the network.

Three caveats qualify this summary. First, the evidence base is dominated by murine and *in vitro* studies. Of the cargoes discussed, only miR-155 currently reaches Tier 4 evidence; miR-690 reaches Tier 3 in mouse but lacks human cohort validation; the remainder sit at Tier 1–2. Donor-side cargo depletion and *in-vivo* physiological-dose validation are essentially absent for Treg-, dendritic cell-, NK-, and neutrophil-EV claims (**Table 1**). Second, apparent contradictions between human cohort studies of Th17/Treg EV ratios in obesity map to isolation method rather than to true biological discordance. Comparing immunoaffinity-captured CD4^+^ EVs across studies that used differential ultracentrifugation versus size-exclusion chromatography reveals consistent biology once the technique is controlled (45). Third, the sub-stoichiometric cargo problem (§2.4) applies with particular force here. Single Treg- or M2-EV-cargo delivery cannot account for population-level metabolic rescue at physiological concentrations; the observed effects require cooperative delivery and likely involve concentration-dependent threshold dynamics that have not yet been formally modeled.

These caveats are not reasons to discount the immune-cell arc. They define the experimental program needed to convert vesiculome-level claims from associational frameworks into mechanistic ones. The bipartite organization developed here — pro-inflammatory and pro-resolving axes whose ratio rather than whose absolute output determines metabolic tone — is the first empirical instantiation in this review of Axiom (iii) (network-balance determinism). §4 develops the reciprocal arc, completing the bidirectional circuit on which the disease-mechanism analysis of §5 (a configurational, not edge-additive, account) depends.

## 4 The metabolic-cell arc of the vesiculome: how metabolic tissues reprogram the immune compartment

**Figure 3:**
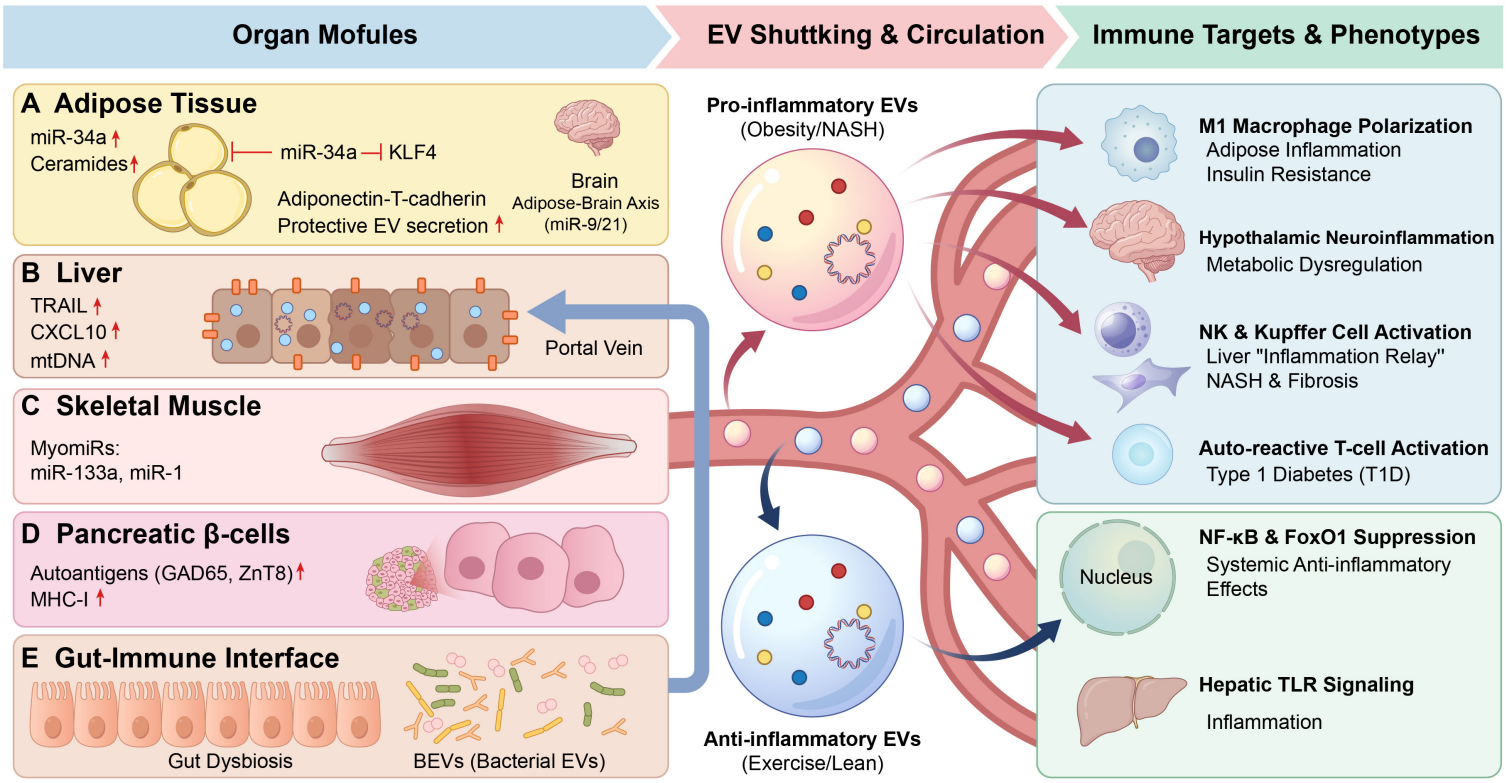
Reverse immunoregulation of the immune system by metabolic-cell-derived EVs. The schematic depicts the five principal “organ modules” through which metabolic tissues actively reshape the systemic immune landscape via EVs. **(A)** Adipose Tissue: Hypertrophic adipocytes secrete EVs enriched in miR-34a and ceramides that suppress KLF4, polarizing macrophages toward the M1 phenotype; protective EV secretion is enhanced through the adiponectin–T-cadherin axis, and miR-9/21-enriched EVs participate in the adipose–brain axis. **(B)** Liver: Lipotoxic hepatocytes release EVs carrying TRAIL, CXCL10, and mtDNA via the portal vein, activating NK cells, Kupffer cells, and hepatic stellate cells (HSCs), thereby establishing a hepatic “inflammation relay” that drives NASH and fibrosis. **(C)** Skeletal Muscle: Exercise-induced myocyte EVs (MyoEVs) carry myomiRs (miR-133a, miR-1) that suppress NF-κB and FoxO1, conferring systemic anti-inflammatory and insulin-sensitizing effects. **(D)** Pancreatic β-Cells: Under stress, β-cells release EVs bearing autoantigens (GAD65, ZnT8) and surface MHC-I, providing the antigenic substrate for autoreactive T-cell activation in type 1 diabetes (T1D). **(E)** Gut–Immune Interface: Intestinal epithelial cell-derived EVs and bacterial extracellular vesicles (BEVs) under dysbiotic conditions traffic LPS/bacterial DNA to the liver via the portal circulation, engaging hepatic TLR signaling. The central “EV Shuttling & Circulation” channel partitions vesicles into pro-inflammatory (obesity/NASH state) and anti-inflammatory (exercise/lean state) populations that converge on distinct immune phenotypes. EV, extracellular vesicle; KLF4, Krüppel-like factor 4; TRAIL, tumor necrosis factor-related apoptosis-inducing ligand; CXCL10, C-X-C motif chemokine ligand 10; mtDNA, mitochondrial DNA; NK, natural killer cell; HSC, hepatic stellate cell; NASH, non-alcoholic steatohepatitis; MyoEV, myocyte-derived EV; myomiR, muscle-specific microRNA; NF-κB, nuclear factor kappa-light-chain-enhancer of activated B cells; FoxO1, forkhead box O1; GAD65, glutamic acid decarboxylase 65 kDa; ZnT8, zinc transporter 8; MHC-I, major histocompatibility complex class I; T1D, type 1 diabetes; BEV, bacterial extracellular vesicle; LPS, lipopolysaccharide; TLR, Toll-like receptor.

This section develops the reciprocal arc of the immune–metabolic vesiculome. Adipocytes, hepatocytes, skeletal myocytes, pancreatic β-cells, and intestinal epithelial cells were once considered passive targets of immune-cell signaling. Accumulating evidence demonstrates the reverse: these metabolic cells actively secrete EVs that deliver lipid mediators, inflammatory ligands, autoantigens, and microbial signals to immune populations, reshaping both the local and systemic immune microenvironment. Combined with §3, this arc closes a bidirectional circuit that is the operational substrate of the vesiculome and the upstream determinant of the disease configurations developed in §5. As in §3, a consistent two-axis pattern emerges. Lipotoxic, stressed, or dysbiotic metabolic cells release pro-inflammatory EVs that polarize macrophages, expand Th17 cells, and break immune tolerance. Healthy or exercised metabolic cells release counter-balancing EVs that sustain immune homeostasis (**Figure 3**; **Table 3**).

### 4.1 Adipocyte-derived EVs as the primary metabolic-cell hub

Adipose tissue is the body’s largest endocrine organ. Adipocyte-derived EVs (AdEVs) therefore occupy a strategic position in systemic immune–metabolic regulation. Under physiological conditions, AdEV cargo supports tissue immune balance. Brown-adipose-enriched miR-99b reaches the liver through the circulation and suppresses hepatic FGF21 expression to maintain glucose homeostasis (46). Adiponectin-loaded EVs, together with the adiponectin–T-cadherin axis that promotes EV secretion from cardiac and vascular tissues, exhibit insulin-sensitizing effects *in vitro* and ameliorate insulin resistance when adoptively transferred to high-fat-diet-fed mice (47, 48).

Obesity disrupts this homeostatic baseline through two convergent routes. Adipocyte hypertrophy generates chronic tissue hypoxia, which upregulates exosome secretion and enriches lipogenic enzymes including acetyl-CoA carboxylase and fatty acid synthase in the AdEV proteome (49). Sustained lipid load triggers endoplasmic reticulum stress in parallel, further remodeling cargo composition (50). The net result is a phenotypic shift from homeostatic messenger to pro-inflammatory effector.

The miRNA content of obese AdEVs is the most direct readout of this reprogramming. Adipocyte exosomes deliver miR-34a to neighboring macrophages. miR-34a engages the KLF4 3′UTR, represses KLF4, and closes the M2 polarization trajectory that KLF4 maintains. The macrophage population then biases toward M1 output with elevated TNF-α and IL-1β secretion (51). A reciprocal arm of the same circuit originates from M1-polarized ATMs, whose miR-155-laden exosomes silence PPARγ in adipocytes and degrade insulin signaling (29). The cargo–effect axes have been validated by reciprocal vesicle transfer between lean and obese animals, in which the donor’s metabolic phenotype tracks with the ATM-EV preparation rather than with the recipient’s prior state. miR-34a currently reaches Tier 3 causal evidence (correlative + reciprocal transfer + donor-side knockout); physiological-dose validation is the remaining gap before Tier 4.

The lipid component of obese AdEVs adds a separate layer of activity. AdEVs are enriched in glycerolipids and free cholesterol and function as a lipase-independent route of lipid release from adipocytes. ATMs internalize these vesicles, and the lipid load is sufficient to drive bone marrow precursor differentiation into ATM-like cells (52). An adipocyte–endothelial vesicle exchange completes the local circuit: endothelial cells transfer caveolin-1-containing EVs to adipocytes and receive vesicles in return, with the exchange tuned by fasting, refeeding, and obesity (53).

Beyond the tissue of origin, AdEVs enter the circulation and exert hormone-like long-range effects. Circulating AdEV levels correlate with insulin resistance severity in obese cohorts, and AdEVs from mesenteric adipose drain through the portal venous system to deposit on hepatic Kupffer cells (54, 55). The central nervous system is another long-range target: nanoscale AdEVs traverse the blood–brain barrier through transcytosis, accumulate in the hypothalamus, activate microglial pro-inflammatory phenotypes, and modulate appetite through mTOR signaling (56). Obesity-associated AdEVs also downregulate cerebrovascular tight junction proteins claudin-5 and occludin, increasing blood–brain barrier permeability and accelerating central neuroinflammation (57). The adipose arc of the metabolic-cell vesiculome is therefore both the largest in volume and the most spatially distributed in reach, making AdEV-cargo profiling a high-priority target for liquid-biopsy biomarker development.

### 4.2 Hepatocyte-derived EVs as a taxonomic family of danger signals

Hepatocytes are central to systemic metabolism. Lipotoxic stress converts these cells into an inflammatory secretory state. EV biogenesis is upregulated and cargo composition is restructured, shifting hepatocyte output from a quiescent baseline to a panel of immune-activating signals (58). Hepatocyte-derived EVs are best understood as a taxonomic family of danger signals, each class engaging different innate immune effectors.

The first class is surface-displayed death ligands. Lipotoxicity activates the DR5–caspase-8–ROCK1 cascade in hepatocytes and triggers release of TRAIL-bearing EVs. These vesicles engage DR5 and recruit RIPK1 in macrophages, inducing IL-1β and IL-6 expression. Pharmacological ROCK1 inhibition reduces serum EV levels and attenuates NASH inflammation in mice, confirming the causal role of this axis (59). TRAIL-EV claims currently sit at Tier 3.

The second class is chemokine-loaded EVs. Lipotoxic hepatocytes release vesicles enriched in CXCL10, which recruit monocytes and pro-inflammatory macrophages to the liver and amplify the inflammatory infiltrate that defines NASH histology (60). The third class is nucleic acid danger signals. Hepatocyte-derived EVs carry mitochondrial DNA fragments and mitochondrial double-stranded RNA. The DNA component activates the cGAS–STING axis in recipient macrophages and induces type I interferon and pro-inflammatory programs; the RNA component engages Toll-like receptor 3 to amplify Th17-type inflammation (33, 61). The fourth class is lipotoxic ceramide-loaded vesicles, in which C16:0 ceramide accumulates through the IRE1α–STARD11 axis and propagates the lipotoxic signal to recipient macrophages and stellate cells (62, 63).

Together these four classes form a coordinated immune-activating panel rather than a list of independent signals. Hepatocyte-derived EVs therefore execute, at the vesicle level, the parenchymal-to-stromal communication that defines the histological transition from simple steatosis to steatohepatitis. The same vesicles enter the circulation, where their cargo profile tracks histological severity in human cohorts. Selective isolation of ASGR1^+^ hepatocyte-derived EVs followed by miRNA profiling distinguishes NAFL from NASH with diagnostic performance exceeding conventional serum ALT and AST measurement (64). Hepatocyte EV cargo is therefore one of the two metabolic-cell EV pools where human cohort evidence has matured beyond Tier 1 correlation into validated stratification utility, anchoring the case for EVs as a clinically actionable liquid-biopsy substrate.

### 4.3 Skeletal myocyte-derived EVs as the exercise-inducible pro-resolving arc

Skeletal muscle releases EVs in response to contraction and exercise. These myocyte-derived vesicles preferentially localize to the liver and contribute to whole-body metabolic protection (65). In obesity, this protective signal is attenuated. The resulting asymmetry—loss of muscle-derived protection combined with gain of adipose-derived pro-inflammatory signal—accelerates breakdown of integrated organ-level regulation.

The miRNA cargo of myocyte-derived EVs reflects this protective identity. Myo-miRs including miR-133a and miR-1 are released into the circulation during exercise and modulate insulin signaling in distal metabolic tissues (66). The protein cargo includes irisin and other exercise-induced myokines that modulate adipose tissue browning and macrophage polarization. The pro-resolving direction of myocyte EVs makes them a candidate biomarker of metabolic fitness and a candidate therapeutic vehicle that recapitulates the systemic benefits of exercise in patients who cannot exercise adequately. The mechanistic biology, however, remains at Tier 1–2 evidence. Cargo-specific genetic depletion in myocytes (e.g., conditional knockout of miR-133a in muscle-specific Cre lines) has not been performed in metabolic disease models, and human cohort validation is limited to small exercise-intervention studies. This gap is a tractable experimental priority and is treated as such in §7.

### 4.4 Pancreatic β-Cell EVs and the stress-induced loss of immune privilege

The pancreatic islet possesses unique immune privilege, and β-cell-derived EVs actively contribute to its maintenance under physiological conditions. β-cell EVs carry islet-specific autoantigens including GAD65, ZnT8, and glucose transporter 2; these molecules are detectable in EV preparations from non-diabetic human islets, and the vesicles participate in antigen-information transfer within the islet microenvironment while remaining under immunological surveillance (67). β-cell EVs also carry immunomodulatory miRNAs including miR-7, miR-21, miR-146, and miR-155 that are selectively sorted and packaged. Under physiological conditions, this miRNA traffic biases the local immune microenvironment toward tolerance (68).

Stress conditions—inflammatory cytokine exposure (IFN-γ + IL-1β + TNF-α), hypoxia, or DNA damage—reverse this homeostatic role. β-cell EV secretion increases approximately fourfold; EV-associated concentrations of β-cell autoantigens (proinsulin, GAD65) rise; and MHC class I molecules are upregulated on the EV surface. These changes provide the molecular substrate for activation of autoreactive CD8^+^ T cells (69, 70). The β-cell vesiculome therefore switches under stress from a tolerogenic signal source to an antigen-presenting node that breaks immune privilege.

This switching property has translational implications in two directions. Diagnostically, β-cell-derived EVs hold promise as early-warning liquid-biopsy biomarkers for type 1 diabetes. β-cell dysfunction and autoantigen release can precede seroconversion of circulating autoantibodies, suggesting that β-cell EV profiling may identify islet injury prior to serological positivity (71). Therapeutically, engineered EVs carrying immune checkpoint molecules including PD-L1 have been used to suppress autoreactive T-cell activation in mouse models of type 1 diabetes (72). Engineered EVs presenting autoantigen peptide–HLA complexes with co-stimulatory or co-inhibitory molecules allow precise regulation of antigen-specific T-cell responses (73). Diagnostic claims currently reach Tier 2 (correlative + reciprocal transfer in human islet preparations); therapeutic claims remain at Tier 2 in murine models only, with no Phase II/III clinical evidence in type 1 diabetes as of late 2024.

### 4.5 Intestinal epithelial and bacterial EVs as the upstream microbial interface

Intestinal epithelial cells sit at the physical interface between the host and the gut microbiota. Their secreted EVs coordinate microbial–mucosal immune interactions, maintain intestinal barrier integrity, and regulate the gut–liver metabolic axis (74). Two distinct vesicle populations operate at this interface and require separate treatment.

Host-derived intestinal epithelial cell EVs transfer antigen information to lamina propria immune cells and participate in tolerogenic immune induction (75). They also carry miRNA and protein cargo that acts on the liver, pancreas, and adipose tissue, participating in interorgan energy metabolism coordination (76). Intestinal EVs are implicated in bile acid signaling through possible modulation of FXR-SHP nuclear receptor pathways, providing an additional regulatory layer over hepatic lipid metabolic homeostasis.

Bacterial extracellular vesicles (BEVs) constitute the second population. BEVs derived from the gut microbiota modulate mucosal immunity through two principal routes. BEVs internalized by intestinal epithelial cells activate NOD1 receptors, triggering NF-κB activation and secretion of IL-6 and IL-8. BEVs penetrate the epithelium directly via M cells or are sampled through dendritic cell transepithelial extensions, contacting lamina propria dendritic cells and modulating their differentiation in a strain-specific manner (77). In obesity, dysbiosis promotes systemic entry of lipopolysaccharide- and bacterial DNA-containing BEVs, which activate the cGAS–STING pathway in hepatic and metabolic tissues and amplify metaflammation. A concomitant reduction in hepatic CRIg^+^ macrophages in obesity further amplifies systemic dissemination of gut-derived BEVs and worsens the metabolic phenotype (78).

This host–microbiota EV crosstalk closes the upstream end of the metabolic-cell arc. Together with the adipocyte, hepatocyte, myocyte, and β-cell vesicles, intestinal and bacterial EVs constitute the full reciprocal half of the immune–metabolic vesiculome. Two summary observations follow. First, every metabolic cell type studied operates a stress-responsive binary switch in EV output—physiological-state EVs sustain immune homeostasis; lipotoxic, hyperglycemic, or dysbiotic-state EVs activate pro-inflammatory immune programs. This binary switch is the metabolic-cell-side manifestation of Axiom (ii) (state-dependent cargo reconfiguration): the same cellular identity produces qualitatively different vesicle outputs depending on metabolic state. Together with the immune-cell-side switch in §3 and the bipartite network organization (Axiom iii), this completes the two-arc topology that Axiom (i) predicts should be resolvable at the level of donor-cell × target-tissue × cargo-class triples. Second, the strength of evidence is highly uneven across the arc. Hepatocyte EVs (TRAIL, CXCL10, ceramide, mtDNA) and adipocyte EVs (miR-34a) reach Tier 3 with limited human cohort validation; myocyte and β-cell EVs sit at Tier 1–2; bacterial EV claims are largely correlative. The §5 disease-mechanism analysis builds on the Tier 3 components and flags the rest as conditional. The methodological priorities required to escalate the conditional claims—single-EV multi-omics, cargo-knockout, and physiological-dose reconstitution—are catalogued in §7.

## 5 Disease as vesiculome network dysregulation: configurations across obesity, MASLD, T2DM, and atherosclerosis

**Figure 4:**
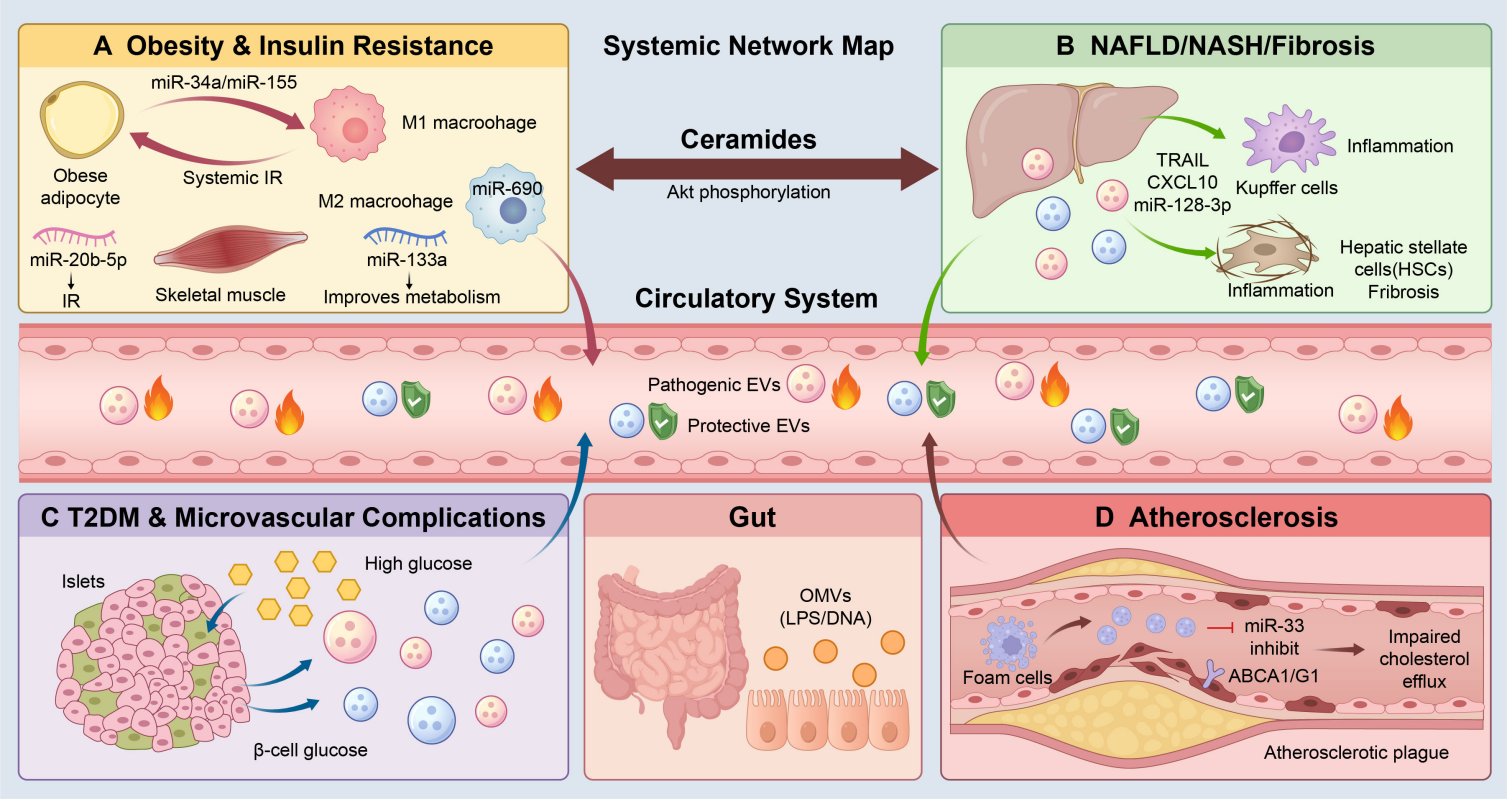
EV-mediated multi-organ crosstalk in the progression of metabolic syndrome. The integrative schematic summarizes the systemic EV-borne communication network underpinning metabolic syndrome. The central circulatory channel carries pathogenic (orange flame) and protective (green shield) EV subpopulations between four disease modules. **(A)** Obesity & Insulin Resistance: A feed-forward circuit between obese adipocytes and M1 macrophages is sustained by exosomal miR-34a (adipocyte→ATM) and miR-155 (ATM→adipocyte), driving systemic insulin resistance. M2-macrophage-derived miR-690 and skeletal muscle-derived miR-133a counterbalance this circuit, while elevated circulating miR-20b-5p impairs insulin signaling in muscle. **(B)** NAFLD/NASH/Fibrosis: Lipotoxic hepatocytes release EVs enriched in TRAIL, CXCL10, and miR-128-3p, activating Kupffer cells and hepatic stellate cells (HSCs) to promote hepatic inflammation and fibrosis. Ceramides bridge this module to **(A)** through Akt dephosphorylation. **(C)** T2DM & Microvascular Complications: High glucose drives β-cells and endothelial cells to release stress EVs that promote monocyte activation, vascular injury, and microvascular complications. **(D)** Atherosclerosis: Foam cell-derived EVs and EV-encapsulated miR-33 suppress ABCA1/ABCG1, impairing reverse cholesterol transport (RCT) and accelerating plaque progression. (Gut): Gut-derived outer membrane vesicles (OMVs) carrying LPS/bacterial DNA enter the portal circulation and feed into modules **(A, B, D)** EV, extracellular vesicle; ATM, adipose tissue macrophage; IR, insulin resistance; PI3K, phosphoinositide 3-kinase; Akt (PKB), protein kinase B; NAFLD, non-alcoholic fatty liver disease; NASH, non-alcoholic steatohepatitis; TRAIL, TNF-related apoptosis-inducing ligand; CXCL10, C-X-C motif chemokine ligand 10; HSC, hepatic stellate cell; T2DM, type 2 diabetes mellitus; OMV, outer membrane vesicle; LPS, lipopolysaccharide; ABCA1/G1, ATP-binding cassette transporter A1/G1; RCT, reverse cholesterol transport.

This section develops the third stage of the four-stage logical chain. §3 and §4 established the two reciprocal arcs of the immune–metabolic vesiculome and their cargo-level mechanisms. §5 traces how systematic dysregulation of this network—not failure of any single edge—produces the clinical phenotypes of obesity, metabolic dysfunction-associated steatotic liver disease (MASLD/NASH), type 2 diabetes mellitus (T2DM), and atherosclerosis. We treat each disease not as an independent entity but as a distinct *configuration* of the same dysregulated vesiculome, exposing shared therapeutic levers and disease-specific signatures (**Figure 4**; **Table 3**). The fifth subsection synthesizes how these configurations converge on a single network failure mode in metabolic syndrome and traces the gut as a shared upstream node.

### 5.1 Obesity and insulin resistance: the founding disease configuration

Chronic adipose tissue inflammation drives obesity-associated metabolic disorders, and vesicle-mediated dialogue between adipocytes and macrophages is the molecular substrate through which this inflammatory state is established. The dialogue operates as a bidirectional feedback circuit rather than a one-way signal. The adipocyte-to-macrophage limb is anchored by miR-34a-bearing exosomes. Adipose tissue macrophages (ATMs) internalize these vesicles, miR-34a silences KLF4, and the macrophage population biases toward M1 polarization (51). The macrophage-to-adipocyte limb completes the loop: M1-ATM exosomes carry miR-155 back to adipocytes, PPARγ is suppressed, and insulin responsiveness is diminished (29). The two limbs amplify each other and progressively recruit additional cell types into the inflammatory state.

This circuit is not unopposed. M2 macrophages introduce a counterbalancing signal through miR-690-loaded EVs that target Nadk and dampen inflammatory drive (30). Progressive deterioration of the M2 compartment during obesity weakens this protective arm, tipping the system asymmetrically toward inflammation. The vesiculome configuration of obesity is therefore not simply “increased pro-inflammatory EV output” but a specific imbalance: the pro-inflammatory arc gains amplitude while the pro-resolving arc loses amplitude. This asymmetry is the network-level explanation for why adipose inflammatory tone becomes increasingly difficult to reverse as obesity advances. miR-155 reaches Tier 4 causal evidence in this configuration (correlative + reciprocal transfer + Mir155^-^/^-^ rescue + physiological-dose validation); miR-34a reaches Tier 3; miR-690 reaches Tier 3 in mouse only.

Obesity-associated insulin resistance disseminates beyond adipose tissue. Circulating EVs are the principal carriers of this dissemination, and three cargo classes contribute through distinct molecular logics. Lipid cargo is the most direct: ceramide activates protein phosphatase 2A, which dephosphorylates Akt and disrupts the PI3K–Akt axis; ceramide also activates PKCζ, which blocks Akt translocation. Both mechanisms reduce GLUT4 plasma membrane recruitment and impair insulin-stimulated glucose uptake in recipient cells (3). The miRNA dimension operates in parallel: obese AdEV miR-34a perturbs KLF4-dependent immune–metabolic homeostasis in macrophages, while miR-155-containing vesicles silence PPARγ across hepatocytes, myocytes, and adipocytes. This multi-tissue PPARγ silencing provides a unified molecular explanation for the cross-tissue character of obesity-related insulin resistance.

Human cohort data have extended this picture beyond mouse models. Serum exosomal miR-20b-5p is significantly elevated in patients with T2DM compared with normoglycemic controls. Transfection of miR-20b-5p into primary human skeletal muscle cells reduces AKTIP protein abundance and impairs insulin-stimulated glycogen synthesis (79). The miRNA content of circulating vesicles therefore tracks metabolic disease status in patients and is functionally coupled to a tissue-level glucose handling defect. Proof-of-principle for organ-level transferability comes from reciprocal vesicle transfer in mice. ATM-EVs from obese donors elevated fasting glycemia, impaired glucose tolerance, and reduced insulin-stimulated Akt phosphorylation in lean recipients; the symmetric experiment in obese recipients of lean-donor vesicles produced corresponding improvement (29). Circulating EVs therefore occupy a dual role in this disease configuration: diagnostic readout of metabolic state and active driver of its dissemination.

The adipose–liver–muscle axis adds organ-level depth to the obesity configuration. Hepatocytes internalize obese AdEVs that carry reduced miR-141-3p; the derepression of PTEN dampens Akt phosphorylation and impairs hepatocellular insulin sensitivity (65). Ceramide species in the same vesicle pool independently inhibit hepatic PI3K–Akt signaling through PP2A activation, elevating glucose output and accelerating *de novo* lipogenesis. Nucleic acid and lipid signals therefore converge on a single signaling axis in the recipient cell. Along the adipose-to-muscle vector, the same pro-inflammatory miRNAs that disrupt adipose immune homeostasis reach skeletal muscle through circulating EVs and compromise oxidative metabolic capacity. Skeletal muscle reciprocates under physiological conditions, releasing EVs that preferentially localize to the liver and contribute to whole-body metabolic protection. Obesity attenuates this protective signal, leaving the asymmetric pattern that characterizes the disease configuration.

### 5.2 MASLD/NASH: the stage-dependent vesiculome configuration

The vesiculome configuration of MASLD evolves with histological stage. The cargo and cell-of-origin profile of circulating EVs is not static; it tracks the transition from simple steatosis through steatohepatitis to fibrosis, providing a window into disease state that is increasingly attractive for non-invasive monitoring (58). In simple steatosis, mild endoplasmic reticulum stress in lipid-laden hepatocytes elevates baseline EV release. The cargo is biased toward lipid metabolism proteins; vanin-1-bearing microparticles dominate the angiogenic dimension; broader immunostimulatory capacity is muted (80). The transition into NASH marks a qualitative escalation. Vesicle output rises more steeply, and cargo undergoes restructuring. C16:0 ceramide accumulates through the IRE1α–STARD11 axis (62, 63). CXCL10 chemokine, TRAIL death ligand, and mitochondrial DNA fragments are released in parallel (59–61). The central event of this transition is the surge in TRAIL-bearing EVs released through DR5–caspase-8–ROCK1 signaling. Macrophage engagement of these vesicles drives IL-1β and IL-6 expression and licenses the hepatic inflammatory response that defines NASH histology (59).

The fibrotic stage brings cellular-origin remodeling. Activated hepatic stellate cells, liver sinusoidal endothelial cells, and infiltrating immune cells each contribute substantial EV output, joining the parenchymal hepatocyte pool that dominated earlier stages. Serum EV concentrations and circulating cell-specific EV signatures track histological fibrosis severity in both animal and human samples, anchoring the case for circulating EVs as non-invasive substrates for fibrosis surveillance (81). The mechanism by which hepatocyte-derived EVs activate hepatic stellate cells operates through three independent cargo routes that converge on the same cellular endpoint. Vesicular CXCL10 binds CXCR3 on stellate cells and triggers their activation. Lipotoxic ceramide species delivered in the same vesicle pool potentiate this activation through stress-signaling cross-talk. Hepatocyte-derived mitochondrial DNA fragments engage Toll-like receptor 9 in stellate cells and amplify pro-fibrogenic gene programs. The cargo redundancy explains why hepatic stellate cell activation in NASH is robust to perturbation of any single pathway.

The vesiculome configuration also involves a gut–liver axis with bidirectional signaling. Bacterial extracellular vesicles from a dysbiotic microbiota enter the portal venous system and deposit on hepatic Kupffer cells, where lipopolysaccharide- and DNA-rich BEV cargo activates cGAS–STING and amplifies hepatic inflammation (78). Hepatocyte-derived EVs in turn modulate intestinal barrier function through gut–liver portal traffic, completing the loop. This bidirectional gut–liver vesicular dialogue is treated in detail in §5.5 because it underlies the systemic dimension of metabolic syndrome more broadly.

The translational maturity of MASLD vesiculome biology is higher than for any other disease in this section. Hepatocyte-specific EVs isolated through ASGR1^+^ immunoaffinity, profiled for miR-122 and miR-192, distinguish NAFL from NASH with diagnostic performance exceeding conventional serum ALT and AST measurement (64). Performance has been validated across two independent cohorts totaling > 300 patients. Hepatocyte EV cargo therefore reaches Tier 4 causal evidence in this disease configuration and has matured into validated stratification utility, anchoring the clinical case for the vesiculome framework.

### 5.3 T2DM: the β-cell-centered vesiculome configuration

The vesiculome configuration of T2DM centers on the islet but extends across the vasculature and the periphery. Within the islet microenvironment, EVs secreted by M1-polarized macrophages—containing TNF-α, IL-1β, and pro-inflammatory miRNAs—act on β-cells to impair insulin gene transcription and glucose-stimulated insulin secretion. The result is an “inflammation → β-cell dysfunction” vesicular circuit whose non-coding RNA regulatory basis has been mechanistically characterized in murine models (70, 82).

Hyperglycemia reshapes endothelial vesicular output and propagates the signal systemically. Under sustained high glucose, endothelial EV release rises and the vesicle surface displays increased phosphatidylserine externalization. The cargo is enriched in pro-inflammatory adhesion molecules ICAM-1 and VCAM-1 and shifts toward miR-503 and miR-155 with concomitant reduction in vasoprotective miR-126. This bidirectional dysregulation impairs VEGF pathway activation and vascular repair, providing the immune–environmental substrate for diabetic vascular complications (83).

The vesiculome configuration of T2DM extends to microvascular complications. The urinary exosomal miRNA profile is significantly altered in diabetic patients. Urinary exosomal miR-130a and miR-145 are upregulated, while miR-155 and miR-424 are downregulated, in type 1 diabetic patients with microalbuminuria—a profile closely associated with early diabetic nephropathy progression (84). Tubular epithelial cell-derived EVs carrying miR-19b-3p are internalized by macrophages, activate M1 polarization, promote renal inflammatory responses, and accelerate kidney injury (85). In the retinal compartment, hyperglycemia induces robust ROS production in retinal pigment epithelial cells and drives release of VEGF-containing exosomes that promote pathological neovascularization in diabetic retinopathy (86). The vesiculome configuration of T2DM is therefore distinguished from that of obesity by the prominence of the β-cell-stress switch (developed in §4.4) and by the propagation of vascular EV dysregulation into target-organ damage. The therapeutic implications follow directly: interventions that restore islet-macrophage vesicular balance and dampen endothelial EV pro-inflammatory output would address the configuration at its hub nodes rather than at peripheral edges.

Translational maturity in T2DM is heterogeneous. Plasma miR-20b-5p elevation as an insulin-resistance biomarker reaches Tier 3 with > 145-patient cohort validation. Urinary EV miRNA panels for diabetic nephropathy reach Tier 2 (correlative in two cohorts). Therapeutic deployment of M2-EV preparations to restore islet immune balance remains preclinical. Engineered β-cell EVs carrying PD-L1 to suppress autoreactive T cells have been demonstrated in murine type 1 diabetes models but have not entered Phase II in either type 1 or type 2 diabetes (72).

### 5.4 Atherosclerosis: the vascular-lipid vesiculome configuration

Foam cell-derived EVs are central drivers sustaining the pro-inflammatory microenvironment within atherosclerotic plaques. Under cholesterol overload, EV secretion by foam cells rises relative to normal macrophages, and the EVs are enriched in oxLDL-derived lipid oxidation products and pro-inflammatory proteins S100A8 and S100A9. Circulating leukocyte-derived EVs are significantly more abundant in patients with atherosclerosis than in healthy controls. These EVs promote migration and adhesion of vascular smooth muscle cells (VSMCs) and contribute to plaque destabilization (87, 88). The chemokines they carry recruit circulating monocytes into plaques; the transfer of pro-inflammatory cargo induces a phenotypic shift of intraplaque VSMCs toward a pro-inflammatory state; matrix metalloproteinase secretion is upregulated; and fibrous cap degradation accelerates plaque progression toward instability.

Platelet-derived microvesicles (PMVs) add a thrombotic-inflammatory dimension. Shear stress at atherosclerotic lesion sites activates platelets, inducing release of PMVs that are surface-enriched with P-selectin, GPIbα, and GPIIb/IIIa and carry arachidonic acid metabolites and activated coagulation factor complexes. Interaction between PMVs and intraplaque macrophages occurs through stable P-selectin/PSGL-1 contact, and bioactive cargo within PMVs activates macrophage pro-inflammatory pathways, facilitating further conversion of macrophages to foam cells (87). This platelet–macrophage vesicular axis is the molecular bridge connecting hemodynamic stress to immune amplification within the plaque.

EVs also impair reverse cholesterol transport, a process essential for resolving lipid accumulation in macrophages. miR-33a and miR-33b act in concert with their host genes SREBP2 and SREBP1 to downregulate ABCA1 and ABCG1, the two ABC transporters required for macrophage cholesterol efflux to ApoA-I and HDL. Loss of these transporters triggers a positive-feedback cycle of cholesterol accumulation and accelerated foam cell formation. In murine models, antagonism of miR-33 upregulates ABCA1/ABCG1 expression, promotes reverse cholesterol transport, and attenuates atherosclerosis progression (89). miR-33 currently satisfies Tier 4 causal evidence in the atherosclerosis configuration.

The atherosclerosis configuration of the vesiculome therefore differs structurally from the obesity, MASLD, and T2DM configurations in two ways. First, the pro-inflammatory arc is dominated by intraplaque cell types—foam cells, platelets, intraplaque immune cells—rather than by adipose, hepatic, or islet hubs. Second, the disease is driven not by failure of a pro-resolving arc but by amplification of a specific pro-inflammatory edge (reverse cholesterol transport impairment) that is mechanistically tractable with miR-33 antagonism. This explains why atherosclerosis has been the most successful therapeutic target of EV-related interventions to date, even though no anti-miR-33 has yet completed Phase III evaluation in humans.

### 5.5 Network-level synthesis: the gut as shared upstream node and the failure mode of metabolic syndrome

The conceptual transition from non-alcoholic fatty liver disease to MASLD underscores the inseparability of hepatic pathology from systemic metabolic abnormalities. Across the four configurations above, EVs interconnect adipose tissue, liver, gut, skeletal muscle, pancreas, and the vasculature, transforming metabolic dysfunction initially confined to individual organs into systemic, synchronized immune–metabolic dysregulation. The balance between protective homeostatic signals and pathogenic inflammatory signals within EV cargo ultimately determines the trajectory of metabolic disease progression.

The adipose–liver–gut triangle illustrates the systemic mechanism. Pro-inflammatory EVs from obese adipose tissue are internalized by hepatocytes, impairing insulin signaling while simultaneously influencing gut microbiota composition and intestinal epithelial barrier function. The dysbiotic intestinal environment reinforces hepatic inflammatory activation through portal EV trafficking. Bacterial EVs carrying lipopolysaccharide and microbial DNA reach hepatic Kupffer cells, activate cGAS–STING and Toll-like receptor signaling, and further amplify the metaflammatory state. A closed inflammatory amplification loop is thereby established. Along the skeletal muscle–liver axis, skeletal muscle passively receives pro-inflammatory EVs from adipose tissue and the liver, resulting in muscle insulin resistance. Regular exercise stimulates increased secretion of protective muscle-derived EVs whose protein cargo preferentially accumulates in the liver, conferring potential protective effects on whole-body metabolism. The vesiculome therefore provides an EV-communication mechanism for the beneficial effects of exercise on MASLD (65).

The gut emerges across all four configurations as the shared upstream node. Obesity, MASLD, T2DM, and atherosclerosis differ in their downstream tissue manifestations but converge upstream on dysbiotic bacterial EV input. The systemic entry of LPS- and bacterial DNA-containing BEVs activates cGAS–STING in hepatic and metabolic tissues; the concurrent reduction in hepatic CRIg^+^ macrophages in obesity amplifies systemic dissemination of these EVs and worsens the metabolic phenotype. The gut–vesiculome interface is therefore the single most consequential therapeutic target identifiable from the network-level synthesis, and dietary, prebiotic, or BEV-targeted interventions warrant prioritization in §6 and §7.

Two integrative observations close this section. First, every disease configuration analyzed here — obesity, MASLD, T2DM, atherosclerosis — exhibits the same general failure mode of the vesiculome: progressive imbalance, loss of the pro-resolving arc and gain of the pro-inflammatory arc, rather than failure of any single edge. This is the empirical content of Axiom (iii) (network-balance determinism, §1) across four independent disease configurations; it converts Axiom (iii) from a theoretical statement into a pattern that recurs at the level of clinical phenotype. Second, the strength of causal evidence varies substantially across configurations. Hepatocyte EV cargo in MASLD and miR-33 in atherosclerosis reach Tier 4. miR-155 and miR-122 reach Tier 4 in their respective configurations. Most other claims sit at Tier 1–3 and require systematic experimental escalation. The translational chapter (§6) builds on the Tier 3–4 components and flags the rest as conditional. The methodological priorities required to escalate the conditional claims are catalogued in §7. Understanding metabolic syndrome as a vesiculome network failure—rather than as a list of organ-specific pathologies—provides the theoretical foundation for the network-level therapeutic strategies treated next.

## 6 Translational applications of the vesiculome: biomarkers, therapeutics, and the manufacturing–regulatory reality

**Figure 5:**
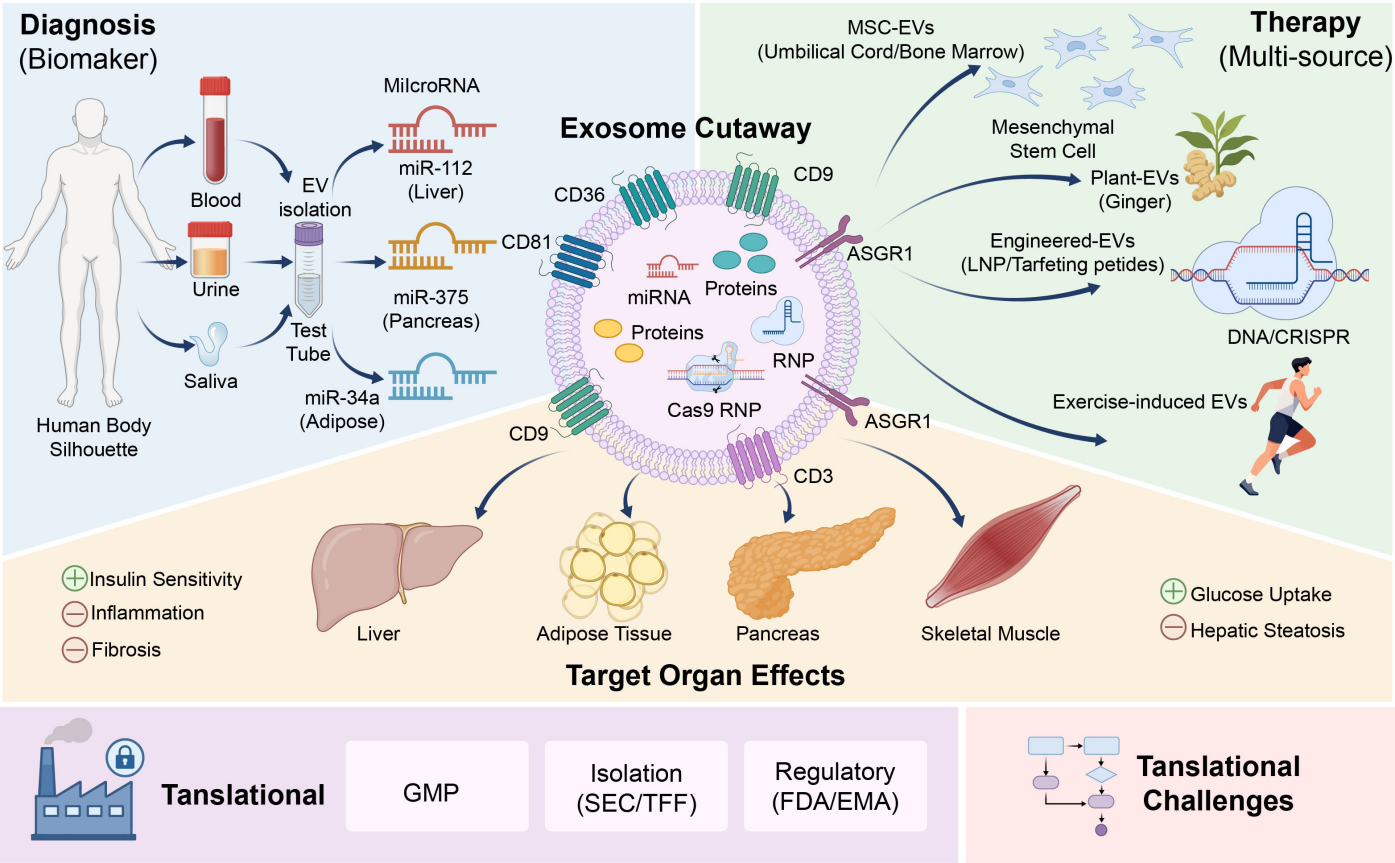
The translational landscape of EVs in metabolic diseases. The schematic illustrates the dual translational identity of EVs as non-invasive biomarkers and versatile therapeutic vehicles. (Left — Diagnosis/Biomarker): EVs are isolated from blood, urine, or saliva (liquid biopsy paradigm). Organ-specific EV miRNAs serve as functional readouts of parental-tissue state: miR-122 (liver/NAFLD), miR-375 (pancreatic β-cell/T2DM), and miR-34a (adipose tissue/obesity-associated insulin resistance). (Center — Exosome Cutaway): The intact phospholipid bilayer encloses miRNA, protein, ribonucleoprotein (RNP), and engineered Cas9–RNP cargo; tetraspanins (CD9, CD81), the scavenger receptor CD36, and the hepatocyte-specific asialoglycoprotein receptor 1 (ASGR1) function as organ-addressing surface determinants. (Right — Multi-source Therapy): Therapeutic EVs are obtainable from mesenchymal stem cells (MSC-EVs from umbilical cord/bone marrow), edible plants (e.g., ginger-derived nanoparticles, PDNVs), engineered platforms (lipid nanoparticle-decorated or targeting peptide-displayed EVs, including CRISPR/Cas9 delivery), and exercise-induced circulating EVs. (Bottom — Target Organ Effects): Therapeutic EVs converge on liver, adipose tissue, pancreas, and skeletal muscle, restoring insulin sensitivity, suppressing inflammation, attenuating fibrosis, enhancing glucose uptake, and reducing hepatic steatosis. (Translational Challenges): Clinical translation requires GMP-grade manufacturing, scalable isolation (SEC/TFF), and a fit-for-purpose regulatory framework (FDA/EMA). EV, extracellular vesicle; CD, cluster of differentiation; ASGR1, asialoglycoprotein receptor 1; RNP, ribonucleoprotein; Cas9, CRISPR-associated protein 9; CRISPR, clustered regularly interspaced short palindromic repeats; MSC, mesenchymal stem cell; LNP, lipid nanoparticle; PDNV, plant-derived nanovesicle; NAFLD, non-alcoholic fatty liver disease; T2DM, type 2 diabetes mellitus; GMP, Good Manufacturing Practice; SEC, size exclusion chromatography; TFF, tangential flow filtration; FDA, U.S. Food and Drug Administration; EMA, European Medicines Agency.

This section develops the fourth stage of the four-stage logical chain. §5 traced how dysregulation of the vesiculome produces the disease configurations of obesity, MASLD, T2DM, and atherosclerosis. §6 evaluates how this same network can be addressed clinically, both as a source of liquid-biopsy biomarkers and as a therapeutic substrate. We apply the four-tier causality framework to every translational claim and distinguish preclinical from human-validated evidence throughout. Five subsections treat (i) liquid-biopsy biomarkers, (ii) native therapeutic EVs, (iii) engineered and mesenchymal stem cell-derived EVs, (iv) the therapeutic-dose reality-check, and (v) manufacturing and regulatory constraints (**Figure 5**; **Table 2**).

### 6.1 EVs as liquid-biopsy biomarkers: cohort-validated stratification

Extracellular vesicles can be collected non-invasively from blood, urine, and saliva, positioning them within the liquid-biopsy paradigm (11). The surface protein composition, lipid content, and intraluminal nucleic acid cargo of EVs reflect the functional state of their parental cells. EVs from key metabolic cell types—pancreatic β-cells, hepatocytes, and adipocytes—undergo substantial changes in abundance and molecular composition under metabolic stress, providing early functional signals of dysregulation (90, 91). Three properties make EVs preferable to soluble-analyte biomarkers in metabolic disease. The phospholipid bilayer protects nucleic acid and protein cargo from degradation by circulating nucleases and proteases, conferring superior stability over cell-free miRNAs (11, 92). Cell-type-specific surface markers enable isolation of tissue-resolved EV subpopulations from a single blood sample. EV cargo is continuously remodeled by donor-cell state in a manner that reproduces the responsiveness of the underlying biology (12). These properties together explain why EV-based liquid-biopsy strategies have reached translational maturity faster than most circulating biomarker platforms.

The clearest example is hepatocyte EV stratification of MASLD. Selective isolation of ASGR1^+^ hepatocyte-derived EVs followed by miR-122 and miR-192 profiling distinguishes simple steatosis from steatohepatitis with diagnostic AUC ≈ 0.84 across two independent cohorts totaling > 300 patients (64). Performance exceeds that of conventional serum ALT and AST measurement, which perform inadequately for histological discrimination. Liver biopsy remains the gold standard but is invasive and unsuited to longitudinal monitoring (93). The hepatocyte EV cargo therefore reaches Tier 4 causal evidence and has matured beyond research utility into the validation phase of biomarker development. In T2DM, plasma exosomal miR-20b-5p is elevated in patients relative to normoglycemic controls and correlates with HOMA-IR; transfection of miR-20b-5p into primary human skeletal muscle cells reduces AKTIP and impairs insulin-stimulated glycogen synthesis (79). The miR-20b-5p signal therefore links the human cohort observation to a tissue-level mechanism. In type 1 diabetes, β-cell-derived EVs carrying miR-375 dynamically reflect β-cell mass and capture information that fasting glucose and HbA1c cannot (94). In diabetic nephropathy, urinary exosomal miR-130a and miR-145 are upregulated while miR-155 and miR-424 are downregulated in patients with microalbuminuria—a profile that precedes overt nephropathy and provides an early non-invasive readout of kidney injury (84).

Three constraints qualify the translational maturity of EV biomarkers. EV abundance and composition are influenced by age, sex, circadian rhythm, exercise, and diet, producing substantial baseline variability that has not yet been mapped through standardized reference intervals (10). Co-occurring metabolic conditions—T2DM with obesity, MASLD with metabolic syndrome—compound EV signatures and obscure disease-specific signals (91). Cross-laboratory baseline coefficients of variation for EV concentration measurements remain in the 30–60% range across MISEV-compliant laboratories. These constraints define the standardization agenda required to convert the current Tier 3–4 biomarker candidates into Phase II/III diagnostic deployment. Recent application of machine learning to multi-omic EV data has yielded encouraging non-invasive diagnostic models for MASLD; integration of single-EV profiling with computational frameworks now provides a path toward population-scale stratification (95).

#### 6.1.1 Human cohort evidence catalogue

The distinction between preclinical and clinically validated EV findings is essential to honest translational appraisal. Robust human evidence currently exists for: (i) ASGR1^+^ EV-derived miR-122 and miR-192 distinguishing simple steatosis from NASH (AUC ≈ 0.84, n > 300, two independent cohorts) (64); (ii) urinary exosomal miR-130a and miR-145 elevation preceding microalbuminuria in type 1 diabetes (case–control, n = 89) (84); (iii) plasma exosomal miR-20b-5p elevation correlating with HOMA-IR in T2DM (n = 145) (79); and (iv) circulating leukocyte-derived EVs elevated in coronary atherosclerosis (multiple cohorts, n > 800) (87, 88). The following remain preclinical only: miR-690 M2-EV therapy (mouse only); engineered PD-L1 EVs for T1D (mouse only); essentially all MSC-EV preparations in metabolic indications (Phase I safety only). **Table 2** catalogues every translational claim in this review by highest evidence tier.

### 6.2 Native therapeutic EVs: cell-of-origin as a pre-configured signal

The therapeutic exploitation of EVs divides into two distinct strategies that warrant separate treatment. Native EVs—harvested from a chosen donor cell type and administered without modification—exploit the natural cargo and homing properties of a physiologically defined vesicle population. Engineered EVs treat the vesicle as a delivery scaffold tailored at the level of surface presentation and luminal cargo. The two strategies carry different risk–benefit profiles, progress on different regulatory timelines, and require different manufacturing infrastructure. This subsection treats native EVs; the next treats engineered and MSC-derived EVs together.

The conceptual basis for native EV therapy is that cellular polarization states are themselves loaded into the secreted vesicle population. Choosing the donor cell selects a pre-configured biological signal whose stoichiometry has been optimized by the donor cell itself rather than by an engineer. Two contexts illustrate the principle. M2-polarized macrophage exosomes loaded endogenously with miR-690 suppress Nadk in recipient cells and restore glucose homeostasis in obese mice without requiring any genetic engineering of the EV population (30). Adipose-derived stem cell exosomes deliver activated STAT3 protein to recipient macrophages, transactivate arginase-1, and bias the local macrophage population toward an anti-inflammatory phenotype. In diet-induced obese animals, this single intervention improves insulin sensitivity by approximately 27.8%, attenuates adipose inflammatory infiltration, and reduces hepatic steatosis—a multi-target profile not replicable with any single recombinant cytokine (32). Both preparations reach Tier 3 causal evidence in mouse models. Neither has been evaluated in humans for metabolic indications.

The translational appeal of native EV preparations lies in their multi-axis activity. A single M2-derived vesicle simultaneously conveys polarization-defining proteins, immunomodulatory miRNAs, and bioactive lipids in proportions that no synthetic cocktail has been able to match. The corresponding limitation is precisely the same multi-axis property: native EV potency assays remain difficult to define, batch-to-batch consistency is hard to verify, and the regulatory pathway for a heterogeneous biological substance remains poorly mapped. Native EV therapy is therefore most realistic for indications where the polarization-defining cell type is itself a recognized therapeutic target and where alternative biologics have failed—an alignment that obesity-associated insulin resistance and MASLD inflammation both satisfy.

### 6.3 Engineered EVs and mesenchymal stem cell-derived EVs

Engineered EVs (eEVs) treat the vesicle as a programmable delivery vehicle. Three engineering dimensions are currently under active development. Surface engineering installs tissue-targeting peptides or antibody fragments to bias biodistribution. Cargo engineering loads defined therapeutic molecules—siRNAs, mRNAs, CRISPR ribonucleoproteins, or small molecules—through electroporation, sonication, or donor-cell transfection. Membrane engineering modifies lipid composition or installs immune-evasion ligands such as CD47-mimetic peptides to extend circulation time. In type 1 diabetes models, engineered EVs carrying PD-L1 suppress autoreactive T-cell activation; engineered EVs presenting autoantigen peptide–HLA complexes with co-stimulatory or co-inhibitory molecules enable precise regulation of antigen-specific T-cell responses (72, 73). These constructs reach Tier 2 evidence in mouse only; no engineered EV has entered Phase II in any metabolic indication.

Mesenchymal stem cell-derived EVs (MSC-EVs) occupy a distinct therapeutic niche. By dissociating the regenerative signal of stem cells from the cells themselves, MSC-EV preparations sidestep the principal liabilities of live-cell therapy—immunogenic mismatch, unpredictable *in vivo* distribution, and residual oncogenic risk of viable transplanted cells—while gaining practical advantages in cold-chain storage, transportation, and unit-dose quality control (96). In T2DM, human umbilical cord MSC-derived exosomes (HucMSC-Exos) act on three distinct nodes of the disease in coordinated fashion. At peripheral tissues, IRS-1 and AKT recover tyrosine-site phosphorylation and GLUT4 reaches the plasma membrane more efficiently. In the liver, glycogen storage capacity is rebuilt. At the islet level, β-cell apoptosis induced by streptozotocin is attenuated and insulin secretory function is preserved (97). A complementary mechanism operates through AMPK-dependent autophagy in hepatocytes, through which HucMSC-Exos additionally improve hepatic glucose and lipid metabolism—a single preparation acting on multiple metabolic axes simultaneously rather than at a single endpoint (98).

The MSC-EV therapeutic spectrum extends to fibrogenic liver pathology. Suppression of hepatic stellate cell activation is the principal anti-fibrotic mechanism, with declining extracellular matrix deposition and slowed fibrosis progression (99). The TGF-β1/Smad signaling axis—the canonical fibrogenic driver in NASH—is antagonized in parallel, and autophagy-related pathways that protect hepatocyte viability are modulated (100). The combination is mechanistically attractive because it simultaneously addresses the cellular effectors of fibrosis (HSCs) and the parenchymal cells at risk of injury (hepatocytes). Clinical-translation evidence remains preliminary but encouraging. Bone marrow MSC-derived exosomes have been administered to critically ill patients with COVID-19 complicated by metabolic inflammation; the treatment was well tolerated and provided indirect safety evidence for EV-based intervention in metabolic inflammatory states (101). Definitive efficacy data await the completion of adequately powered randomized controlled trials in metabolic disease populations, which are not yet available.

#### 6.3.1 Plant-derived nanovesicles: a methodological cautionary note

Plant-derived nanovesicles (PDNVs) have attracted attention for natural abundance, low cost, and oral-route compatibility. Ginger-derived exosome-like nanoparticles carry mdo-miR7267-3p and modulate the gut microbiota through Lactobacillaceae uptake, promoting indole-3-carboxaldehyde production and AHR-mediated IL-22 secretion in mouse colitis models (102, 103). However, the central translational claim of cross-kingdom plant-miRNA regulation of mammalian cells has not been reproducibly demonstrated in independent laboratories. Recent attempts to replicate the original cross-kingdom miRNA transfer findings have largely failed, and the methodological standards for PDNV preparations remain non-uniform (104). PDNVs therefore warrant ongoing investigation but should be treated as a methodological cautionary note rather than as a translational platform until cross-kingdom claims are reproducibly substantiated and PDNV-specific nomenclature and minimum characterization standards are established. The current evidence sits at **Tier 1** and does not support imminent clinical translation in metabolic indications.

### 6.4 Therapeutic dose reality-check: the sub-stoichiometric and supraphysiological problem

A persistent constraint on EV-therapeutic translation has received insufficient explicit attention: nearly all preclinical EV-therapy studies administer doses that exceed physiological circulating EV concentrations by 100- to 10,000-fold. Across the literature, doses span four orders of magnitude (10^8^ to 10¹² particles per mouse) with no consistent quantification metric. Particle number, total protein content, and parental-cell number are used interchangeably, rendering cross-study dose comparisons effectively impossible. A systematic review of EV clinical trials through end-2023 found that most early-phase studies did not perform systematic potency assessment of EV products prior to administration, and that variation in administration routes (intravenous, intraperitoneal, oral, inhaled) and dosing regimens severely limited cross-study comparability (105).

Three consequences follow from this dose reality. First, “therapeutic efficacy at supraphysiological dose” is an unreliable indicator of physiological mechanism. The biological pathway engaged at 10¹² particles/mouse may differ qualitatively from the pathway engaged at endogenous concentrations. Second, the sub-stoichiometric cargo problem (§2.4) applies with particular force at physiological doses: if the average copy number of any individual miRNA per EV is below one, then population-level effects require cooperative delivery across many vesicles, and dose–response relationships are unlikely to be linear in the relevant biological range (106). Third, dose-finding studies for EV therapeutics will require explicit calibration against endogenous EV concentrations rather than against *in-vitro* EC_50_ values—a calibration that the current preclinical literature has largely skipped. The minimum standard the field should adopt is threefold: report particles/mL, total protein, and donor-cell-equivalent doses concurrently; calibrate doses against measured endogenous concentrations rather than arbitrary *in-vitro* EC_50_; and include a low-dose arm at or below the physiological range to test whether mechanism is dose-dependent. Where these standards are not met, therapeutic efficacy claims should be presented as proof-of-principle rather than as evidence of native EV function. This reality-check applies to native, engineered, MSC-derived, and plant-derived EV preparations alike.

### 6.5 Manufacturing, safety, and regulatory constraints

The path from a promising preclinical EV preparation to a marketable therapeutic is constrained by three categories of challenge that currently lack mature solutions: manufacturing scalability, safety evaluation, and regulatory classification.

Producing therapeutic-grade EVs at clinically relevant scale with Good Manufacturing Practice (GMP) consistency is the principal bottleneck of the field. Requirements span donor cell selection and characterization, culture system design, downstream purification chemistry, and continuous quality control across production batches (107). Standard two-dimensional culture systems generate vesicle quantities that fall an order of magnitude short of the per-patient doses inferred from preclinical efficacy. Higher-output platforms—hollow-fiber bioreactors and three-dimensional spheroid cultures—have raised yields by approximately 50- to 100-fold, but preserving cargo composition and per-batch biological activity through scale-up remains an open technical problem (108). On the purification side, tangential flow filtration combined with size-exclusion chromatography is consolidating as a GMP-compatible default workflow, though no consensus protocol has yet been formally endorsed.

EV preparations require a safety assessment broader than that applied to most conventional biologics because the active substance is a population rather than a single molecular species. Biodistribution studies must characterize off-target accumulation and assess the biological consequences of EV cargo internalized by non-target tissues. For allogeneic preparations and engineered EVs, repeat-dose immunogenicity must be evaluated under ICH guidance, with attention to whether neutralizing antibody responses develop against engineered surface ligands. CRISPR-Cas9-loaded EVs add a layer of regulatory scrutiny: genome-wide off-target editing must be characterized in cell types representative of *in-vivo* biodistribution, not merely in donor cell lines (109).

Regulatory classification frameworks remain incomplete. In the United States, the FDA assigns EV products to either the HCT/P category (21 CFR Part 1271) or to biologics regulation (21 CFR Part 351), with the threshold determined by origin and degree of manipulation; preparations involving “more than minimal manipulation” are generally directed toward investigational new drug submission. In Europe, the EMA classifies EVs within the Advanced Therapy Medicinal Products (ATMP) framework, with engineered EVs assigned to gene therapy medicinal product or somatic cell therapy medicinal product subcategories according to the extent of genetic modification (110). The 2023 FDA draft guidance on exosome-based oncology products represents an early step toward dedicated EV regulation, but a fully integrated regulatory regime for therapeutic EVs has not yet emerged in any major jurisdiction. Regulatory science must therefore advance in parallel with technological development if institutional barriers are not to slow the clinical adoption of EV-based medicines (28, 111).

The translational maturity of the four EV-therapeutic modalities is therefore heterogeneous. EV-based liquid-biopsy biomarkers have reached the validation phase in MASLD and the discovery phase in T2DM and diabetic nephropathy. Native EVs and MSC-EVs have completed Phase I safety evaluation in COVID-19 metabolic inflammation but await Phase II/III in metabolic disease. Engineered EVs and PDNVs remain preclinical. The structured knowledge-gap analysis in §7 enumerates the specific experiments, standardization steps, and cohort designs required to advance each modality, and the conclusion (§8) synthesizes how the vesiculome framework reshapes the translational agenda from cargo enumeration toward network-level intervention.

## 7 Knowledge gaps, controversies, and priority research directions

This section translates the diagnostic critique applied throughout §3–§6 into a structured research agenda. We organize the field’s unresolved problems into three categories—foundational, causal, and translational—and close with a priority-directions framework that specifies the experimental program required to advance the vesiculome from a conceptual claim into an operationally testable construct. The structure mirrors the four-tier causality framework introduced in §1 and used throughout. Foundational gaps constrain the strength of any Tier 1 observation. Causal gaps separate Tier 1–2 from Tier 3–4 evidence. Translational gaps separate Tier 4 preclinical evidence from human deployment. Priority directions specify how to close all three layers in parallel.

### 7.1 Foundational gaps: biology, methodology, and nomenclature

The foundational layer concerns claims that hold regardless of disease context: what an EV is, how it can be reliably identified, and how its cargo can be measured at biologically meaningful resolution. Three foundational gaps recur across every section of this review and define the methodological ceiling of the field.

The first foundational gap is EV heterogeneity at sub-population resolution. Conventional EV isolation produces a population average that conceals substantial sub-population diversity in size, surface marker composition, cargo content, and biological function. Single-vesicle approaches—single-particle interferometric reflectance imaging, multiplexed flow cytometry, single-EV miRNA-seq, and cryo-electron tomography—have begun to dissolve this average into its constituent sub-populations, revealing that vesicles bearing CD9, CD63, and CD81 represent partially overlapping rather than identical sets, and that exomeres and supermeres operate as distinct extracellular communication classes outside the canonical EV taxonomy (18, 26). The MASEV multiplexed single-EV platform and cryo-TEM computational imaging are emerging as practical tools for resolving sub-population structure (112). Until these methods become routine, all population-averaged claims must be treated as candidate signals requiring sub-population validation.

The second foundational gap is isolation and characterization standardization. EV isolation methods are not interchangeable. Differential ultracentrifugation, size-exclusion chromatography, polymer precipitation, immunoaffinity capture, and microfluidic approaches each enrich different sub-populations, and the resulting baseline heterogeneity is the single largest source of cross-study discordance in this field. MISEV2018 and MISEV2023 codify minimum reporting standards and have substantially improved consistency in published work (6). Methodological heterogeneity nonetheless persists across clinical laboratories. Cross-laboratory baseline coefficients of variation for EV concentration measurements remain in the 30–60% range even among MISEV-compliant centers (113, 114). Apparent biological discordance between human cohort studies—Th17/Treg EV ratios in obesity being a representative case (§3.5)—frequently maps to isolation method rather than to true biology. The field’s next standardization advance must therefore extend beyond minimum reporting to harmonization of isolation methods themselves, ideally through cross-laboratory benchmarking exercises against shared reference materials.

The third foundational gap is *in-vivo* tracking. Establishing where therapeutic or endogenous EVs go and how long they persist is currently constrained by fluorescent-labeling artefacts (free dye redistribution that mimics vesicle uptake) and by limited sensitivity for endogenous-dose conditions. Labeling human placenta-derived mesenchymal stem cell EVs with the aggregation-induced emission luminogen DPA-SCP has enabled real-time quantitative tracking of EV accumulation at liver injury sites for up to 7 days in murine acute liver injury models (115). Multimodal imaging that combines optical imaging with PET/SPECT or MRI can provide quantitative spatial information about EV transit between metabolic organs (116, 117). However, these approaches remain restricted to administered therapeutic EVs at supraphysiological doses and have not been validated for endogenous-EV tracking at physiological concentrations. Unified *in-vivo* EV tracking standards—including dye-control protocols, dose calibration, and matched native-EV comparators—are required before therapeutic-EV biodistribution can be characterized at the level of regulatory expectation.

A fourth foundational concern deserves explicit attention: nomenclature. The boundaries between small EVs, microvesicles, exomeres, supermeres, plant-derived nanovesicles, and bacterial outer membrane vesicles are inconsistently drawn across the literature. PDNVs are variously described as “exosome-like nanoparticles,” “nanovesicles,” or “microvesicles” with no agreed-upon biogenesis criteria (118, 119). The MISEV consensus has resolved much of this for animal EVs but does not extend to plant-derived or bacterial vesicles, where minimum characterization standards specific to each class are needed. Without such standardization, the literature will continue to accumulate non-comparable findings, and Tier 4 causal claims will remain difficult to validate across systems.

### 7.2 Causal gaps: correlation, stoichiometry, and bioavailability

The causal layer concerns claims about whether EV cargo *does* the work attributed to it. §1 established the four-tier framework (Tier 1 correlative; Tier 2 reciprocal transfer; Tier 3 donor-side genetic knockout; Tier 4 *in-vivo* physiological-dose validation). §3–§6 applied this framework to every cargo claim. Three persistent causal gaps emerge from that exercise.

The first and most general causal gap is correlation versus causation. The miRNA and protein composition of circulating EVs is substantially altered in metabolic disease states; whether these alterations are functional drivers or coincident biomarkers remains incompletely established for most cargoes (10, 91). Of the dozens of cargoes implicated in metabolic disease across §3–§5, only miR-155, miR-122, miR-690, miR-33, and a small number of others currently reach Tier 4. Most candidate biomarker miRNAs and most candidate therapeutic targets remain at Tier 1–2 and have not been subjected to donor-side cargo depletion and physiological-dose reconstitution. Establishing causality requires integrated genetic knockout and knock-in models targeting specific cargo molecules, combined with multi-level functional validation in recipient cells, converging on the same biological phenomenon from multiple angles (90).

The second causal gap is sub-stoichiometric cargo and the cooperative-delivery problem. The average copy number of any individual miRNA per single EV is sub-stoichiometric; for most reported miRNAs, less than one copy per vesicle on average (7). This single fact has three consequences that the field has been slow to integrate. The dominant mental model of “one vesicle delivers one functional dose of cargo to one recipient cell” is mechanistically untenable for miRNA-based signaling. Biological effect at the population level must arise from cooperative delivery across many vesicles, plausibly via locally elevated cargo concentration at uptake hot-spots or via repeated dosing over hours to days. Most importantly, this makes physiological-dose validation the critical missing experiment for nearly every causal claim. Therapeutic doses delivered as bolus intravenous administration in murine models routinely exceed any plausible endogenous concentration by 100- to 10,000-fold (§6.4). Resolution of this constraint demands single-vesicle multi-omics with absolute quantification and matched *in-vivo* physiological-dose reconstitution; without this, Tier 2/3 evidence cannot be advanced to Tier 4.

The third causal gap is endolysosomal escape and functional bioavailability. Whether EV cargo internalized by recipient cells reaches a functional intracellular compartment, rather than terminating in lysosomal degradation, is rarely measured directly. Functional validation experiments demonstrate that miR-690 from M2 macrophage exosomes is efficiently internalized by insulin target cells following intravenous administration to obese mice and suppresses inflammation through Nadk gene targeting (30). However, single-particle real-time tracking of cargo escape remains technically challenging, and the proportion of internalized vesicles that achieve productive cytosolic delivery is not known for most cargo–recipient pairs. Some investigators contend that proteins and miRNAs detected in circulating EVs are largely in a degraded state and may not represent functionally active signaling molecules (91, 112).This is the central live controversy of the field and is closely coupled to the sub-stoichiometric problem above. Methods that visualize endolysosomal escape at single-particle resolution—pH-sensitive reporters and cargo-tagged proximity ligation—are now emerging and constitute a high-priority methodological direction.

A fourth causal gap warrants mention: disease-stage-dependent functional switching. EV function may shift substantially as disease progresses. During early diet-induced obesity (4 weeks of high-fat diet), hepatocyte-derived exosomes enriched in miR-3075 improve insulin sensitivity in adipose tissue and skeletal muscle through FA2H targeting; in chronic obesity (16–18 weeks), exosomes from the same cell type instead activate pro-inflammatory macrophages and promote systemic insulin resistance (120). This “functional switch” suggests that EV activities are subject to temporal window effects, but systematic longitudinal data defining these windows—and their generalizability across T2DM, MASLD, and atherosclerosis—are currently absent. Static, cross-sectional vesiculome profiling will systematically miss switching behavior of this kind. Time-resolved sampling along disease natural history is therefore required for accurate causal inference.

### 7.3 Translational gaps: human validation, standardization, and regulation

The translational layer concerns claims about whether preclinical findings can be deployed in humans. Three translational gaps recur across the modalities reviewed in §6.

The first translational gap is human–murine discordance. The vast majority of mechanistic conclusions in the EV–metabolic-disease literature derive from murine models and *in-vitro* studies. Human cohort validation exists for a small set of biomarker claims—ASGR1^+^ EV miR-122/miR-192 in MASLD, urinary miR-130a/miR-145 in diabetic nephropathy, plasma miR-20b-5p in T2DM, leukocyte EVs in atherosclerosis—but extends to essentially no therapeutic claim in metabolic indications. Murine-to-human discordance is not a peripheral concern. Murine EV cargo composition, isolation behavior, and biodistribution differ systematically from human counterparts, and the genetic homogeneity of standard mouse strains overestimates the consistency of vesiculome configurations observed across genetically diverse human populations. Tier 3 murine evidence is therefore insufficient for human translational claims and must be supplemented by human-specific cohort validation at minimum.

The second translational gap is the scarcity of standardized human cohorts. Current EV research in metabolic disease is predominantly cross-sectional or reliant on animal models, with prospective longitudinal cohort data markedly scarce. Standardized cohorts spanning the full natural history of metabolic disease—from pre-metabolic-syndrome to confirmed T2DM diagnosis and the emergence of complications—combined with dynamic multi-omics EV profiling, constitute the evidence base necessary for establishing EV biomarker reference intervals and validating predictive models (90). The MASLD field has the highest current priority: existing non-invasive diagnostic tools have limited accuracy, and selective hepatocyte EV isolation combined with miRNA profiling substantially improves diagnostic performance for MASLD staging (64, 121). Such cohorts must also account systematically for the confounding effects of gut microbiota, genetic background, circadian rhythm, dietary patterns, and co-occurring conditions, and should promote standardized cross-center data sharing through pre-registered protocols.

The third translational gap is dose, route, and potency-assay heterogeneity in EV clinical trials. Existing preclinical and early-clinical studies employ EV dosing regimens that vary by orders of magnitude, with inconsistent quantification metrics: particle number, total protein content, and parental cell number are used interchangeably (105). A systematic review of EV clinical trials through end-2023 found that most early-phase studies did not perform systematic potency assessment of EV products prior to administration, and that significant variation in administration routes severely limits the comparability and generalizability of findings. The therapeutic-dose reality-check framework in §6.4 specifies the minimum reporting standard required to escape this state.

A fourth translational concern is regulatory ambiguity. The FDA’s HCT/P versus biologics distinction and the EMA’s ATMP classification create different regulatory pathways for what may be equivalent products in different jurisdictions, complicating multinational trial design (110). The 2023 FDA draft guidance on exosome-based oncology products represents an early step toward dedicated EV regulation, but a fully integrated regulatory regime for metabolic-disease EV therapeutics has not yet emerged. Regulatory science must therefore advance in parallel with technological development through continuing dialogue between the ISEV standardization community and the principal regulatory agencies.

### 7.4 Ongoing controversies across the field

Several controversies warrant explicit acknowledgment because their resolution will reshape the framework developed in this review.

The first is the biological activity of EV cargo at physiological doses. Whether internalized cargo escapes endolysosomal degradation to exert biological activity at endogenous concentrations remains contested (§7.2 above). Single-particle level real-time tracking is technically challenging, and the field will need to resolve this controversy before any miRNA-based EV therapeutic reaches Phase III in metabolic indications.

The second is the cross-kingdom regulatory activity of plant-derived nanovesicle miRNAs. PDNVs lack unified nomenclature, and structurally similar entities are variously described across the literature. The influence of plant cultivar, growing conditions, and processing methods on PDNV composition is difficult to standardize. Independent replications of cross-kingdom miRNA transfer claims have failed, and the central translational premise of PDNV-based therapeutics remains contested (118, 119, 122). §6.3 has correspondingly downgraded PDNVs to a methodological cautionary note. Resolution requires dedicated nomenclature frameworks and minimum characterization standards specific to plant vesicles, distinct from those applied to animal EVs.

The third is whether therapeutic-EV efficacy at supraphysiological doses reflects native EV biology or a pharmacological effect unique to bolus administration. The dose reality-check developed in §6.4 makes this an empirically tractable question through low-dose-arm inclusion in future preclinical and early-phase studies.

### 7.5 Priority research directions

The structured agenda required to close the gaps catalogued above operates along five interlocking dimensions. We frame each as a specific experimental program rather than as a topic area, in keeping with the operational definition of the vesiculome adopted in §1.

The first priority is single-EV multi-omics integration. Resolving sub-population heterogeneity, sub-stoichiometric cargo distribution, and disease-stage-dependent cargo switching requires integration of single-EV proteome, transcriptome, and lipidome measurements at single-particle resolution (112, 123, 124). Establishing standardized analytical workflows for single-EV multi-omics—particularly through MASEV-class platforms combined with cryo-electron tomography—is an essential prerequisite for advancing Tier 2/3 evidence to Tier 4 and for distinguishing pro-inflammatory from pro-resolving vesicle sub-populations within the mixed vesiculome.

The second priority is *in-vivo* EV tracking at physiological resolution. Real-time monitoring of endogenous EV transit between immune and metabolic organs—not only of therapeutic preparations at supraphysiological doses—requires unified dye-control protocols, dose-matched comparators, and multimodal imaging that combines optical with PET/SPECT or MRI modalities. This program is foundational for both therapeutic biodistribution claims and endogenous-vesiculome dynamics.

The third priority is multi-organ organoid-on-chip platforms {Ronaldson-Bouchard, 2022 #716}. Multi-organ microfluidic systems enable physiological reconstruction of inter-organ vesiculome communication—liver, adipose, pancreas, and gut connected through perfused channels and coupled with co-cultured immune populations. A kidney–liver organoid chip has been used to evaluate biodistribution and therapeutic effects of MSC-derived Evs (125). Integration of macrophages and other immune cells into co-culture systems enables the simulation of metabolic-organ–immune-system interactions on a single platform (126). Organoid-on-chip systems provide the *in-vitro* context with the highest current physiological relevance for testing vesiculome-level hypotheses that cannot be addressed in cross-sectional human cohorts.

The fourth priority is longitudinal human cohorts. Prospective cohort studies spanning the natural history of metabolic disease, combined with dynamic multi-omics EV profiling and standardized cross-center data sharing, are the indispensable evidence base for establishing EV biomarker reference intervals and validating predictive models. The MASLD field is the immediate priority given the existing translational maturity of hepatocyte EV stratification (64, 121), but parallel cohorts in T2DM, obesity, and atherosclerosis are equally needed.

The fifth priority is AI-assisted vesiculome analysis. Machine learning algorithms offer substantial advantages in processing high-dimensional EV multi-omics datasets (127). Computational frameworks integrating deep proteomic data can systematically screen thousands of candidate molecules to identify EV surface marker panels combining diagnostic performance with analytical feasibility (128). The integration of single-EV multi-omics with longitudinal cohort data and AI-driven feature extraction is the natural endpoint of the priority program; it converts the vesiculome from a topology to a computable predictive model.

A sixth direction is the gut-microbiota–vesiculome triangle. Gut microbiota-derived EVs, host metabolic tissues, and the microbiota itself together constitute a bidirectional signaling network that governs metabolic homeostasis. High-fat-diet-induced dysbiosis promotes systemic entry of LPS- and bacterial DNA-containing microbial EVs, activating cGAS/STING and inducing metabolic-organ insulin resistance; concurrent reduction of hepatic CRIg^+^ macrophages amplifies this pathological process (78, 129). The mechanisms by which dietary patterns modulate metabolic disease through microbiota EV reconfiguration represent a translational entry point of high value, demanding interdisciplinary research integrating metagenomics, EV multi-omics, and *in-vivo* functional validation.

These six directions are not independent. Each requires the others—single-EV multi-omics is meaningless without standardized cohorts; cohorts are uninterpretable without AI-assisted analysis; organoid-on-chip platforms require single-EV multi-omics for read-out. The integrated agenda is itself a vesiculome-level statement: the network can only be understood through coordinated, network-level methodology. The Conclusion (§8) synthesizes how the framework developed in this review reshapes the research priorities, the translational pipeline, and the clinical horizon of EV biology in metabolic disease.

## 8 Conclusion

### 8.1 What the vesiculome framework adds

This review has argued that extracellular vesicles are best understood not as a list of cargo–phenotype pairs but as an integrated information layer of immune–metabolic crosstalk. The vesiculome — defined operationally by the three axioms set out in §1: (i) the network is addressable at the resolution of donor cell × target tissue × cargo class; (ii) cargo composition tracks donor-cell metabolic state predictably; (iii) the pro-inflammatory/pro-resolving balance — not any single edge — determines the organismal metabolic phenotype — reframes EV research as a question of network topology and node hierarchy rather than of cargo enumeration.

Network topology becomes the unit of analysis. §3 and §4 established that the immune-cell arc and the metabolic-cell arc of the vesiculome operate as reciprocal half-circuits whose balance—rather than the absolute output of either—determines metabolic phenotype. §5 traced how obesity, MASLD, T2DM, and atherosclerosis arise as distinct *configurations* of the same dysregulated network rather than as four independent diseases. This framing predicts that interventions targeting hub nodes (M1/M2 macrophage polarization, hepatocyte stress switching, β-cell immune privilege) will outperform interventions targeting peripheral edges (individual circulating miRNAs), and it provides a testable rationale for the network-level therapeutic strategies developed in §6.

Evidence quality becomes transparent at the level of every claim. The four-tier causality framework introduced in §1 has been applied throughout. Of the dozens of EV cargoes implicated in metabolic disease, only miR-155, miR-122, miR-690, and miR-33 currently reach Tier 4. The majority of candidate biomarkers and therapeutic targets remain at Tier 1–2 and require systematic experimental escalation. Treating evidence strength as a property of every claim—rather than as a methodological footnote—forces honesty about what is known, what is hypothesized, and what is currently untestable.

Foundational constraints become unavoidable. The sub-stoichiometric cargo problem, the therapeutic-dose reality-check, the murine–human discordance, and the isolation-method heterogeneity are not peripheral methodological concerns. They define the ceiling of what the current literature can claim and the experimental program required to raise that ceiling. The vesiculome framework makes these constraints structural rather than incidental.

### 8.2 Immune–metabolic crosstalk as a vesiculome phenomenon

EV-mediated immune–metabolic communication is inherently bidirectional. Adipose tissue-derived EVs alter macrophage reverse cholesterol transport gene expression and modulate macrophage phenotype; activated immune cells release EVs that feed back onto metabolic tissues and exacerbate lipid metabolic dysfunction (130). This bidirectional crosstalk produces the self-reinforcing pathological circuits underlying the chronicity and poor reversibility of established metabolic disease. The immunologic and metabolic dimensions of metaflammation are not parallel processes that happen to be associated. They are two faces of a single vesiculome program in which donor-cell identity, polarization state, and metabolic context are transmitted as a structured package.

Metabolic disease is fundamentally a disorder of multi-organ functional coordination rather than isolated organ pathology. Under physiological conditions, adipose tissue, liver, skeletal muscle, pancreatic islets, and gut continuously exchange metabolic status signals through EV-based networks, collectively maintaining systemic energy homeostasis. Under pathological conditions, global dysregulation of this network—rather than any single aberrant EV subpopulation—drives the transition from localized metabolic compensation to systemic decompensation. Hepatocytes under lipotoxic stress release saturated fatty acid-enriched small EVs that activate hepatic Kupffer cells via TLR4 signaling; the resulting inflammatory mediators impair hepatocyte insulin signaling, establishing a self-amplifying immune–metabolic feedback loop (131). EV-mediated signaling between islet cells and immune cells has been implicated in the pathogenesis of both type 1 and type 2 diabetes (132). The cascade dysregulation of multi-organ EV networks constitutes the molecular basis for the multi-morbidity pattern of metabolic syndrome and explains its tendency toward systemic progression. Therapeutic strategies targeting single metabolic endpoints are inherently limited in their capacity to disrupt these pathological networks, providing a compelling rationale for developing system-level interventions.

### 8.3 Translational outlook

The translational maturity of the vesiculome differs sharply across the modalities reviewed in §6. EV-based liquid-biopsy biomarkers have reached the validation phase in MASLD, with ASGR1^+^ hepatocyte-derived EVs profiled for miR-122 and miR-192 distinguishing simple steatosis from steatohepatitis at clinically actionable performance (64). Plasma miR-20b-5p in T2DM and urinary EV-miRNA panels in diabetic nephropathy have entered cohort-level validation (79, 84). These signals justify continued investment in standardized large-cohort prospective studies. Native EV therapeutics and mesenchymal stem cell-derived EVs have completed Phase I safety evaluation in adjacent indications but remain preclinical for metabolic disease (101). Engineered EVs and plant-derived nanovesicles remain entirely preclinical, with the cross-kingdom miRNA premise of PDNV-based therapeutics currently contested by independent replication failure.

Three translational priorities follow from this evidence map. First, MASLD biomarker validation should be the immediate near-term focus, given the existing translational maturity and the urgent clinical need for non-invasive histological stratification. Second, native and MSC-EV preparations should advance to Phase II trials in metabolic indications with the dose-calibration framework developed in §6.4, allowing direct interrogation of whether observed efficacy reflects native vesiculome biology or supraphysiological pharmacology. Third, the engineered EV field requires consolidation around a small number of well-characterized hub-targeting constructs (PD-L1 EVs for T1D immune tolerance; tissue-targeted miR-33 antagonist EVs for atherosclerosis), tested with appropriate biodistribution and immunogenicity profiling, before broader expansion is justified.

The integrated research agenda specified in §7—single-EV multi-omics, *in-vivo* tracking at physiological resolution, multi-organ organoid-on-chip platforms, longitudinal human cohorts, AI-assisted vesiculome analysis, and the gut–microbiota–vesiculome triangle—is itself a vesiculome-level statement. The network can only be understood through coordinated, network-level methodology. No single approach—biomarker development, mechanistic discovery, or therapeutic engineering—is sufficient on its own. Combined coordination across the agenda is what converts the vesiculome from a conceptual framework into an operationally testable research program.

### 8.4 Closing statement

The conceptual transition of extracellular vesicles from cellular waste to active signaling nodes is now complete. The field’s next transition—from cargo enumeration to network-level understanding—is the conceptual contribution this review aims to advance. Repositioning EVs as the vesiculome operating as the information layer of immune–metabolic crosstalk provides both a theoretical framework and a falsifiable experimental program for next-generation precision diagnosis and intervention in metabolic disease. The metabolic disease burden continues to rise faster than therapeutic innovation. Whether the vesiculome framework matures into a clinical reality will depend on the rigor with which the foundational, causal, and translational gaps catalogued in §7 are systematically closed. The trajectory is tractable. The agenda is specified. The work now is execution.
